# The unfolded protein response is activated in the olfactory system in Alzheimer’s disease

**DOI:** 10.1186/s40478-020-00986-7

**Published:** 2020-07-14

**Authors:** Helen C. Murray, Birger Victor Dieriks, Molly E. V. Swanson, Praju Vikas Anekal, Clinton Turner, Richard L. M. Faull, Leonardo Belluscio, Alan Koretsky, Maurice A. Curtis

**Affiliations:** 1 Department of Anatomy and Medical Imaging and Centre for Brain Research, Faculty of Medical and Health Science, University of Auckland, Private Bag 92019, Auckland, New Zealand; 2 Laboratory of Functional and Molecular Imaging, National Institute of Neurological Disorders and Stroke, National Institutes of Health, Bethesda, MD 20892 USA; 3 Department of Anatomical Pathology, LabPlus, Auckland City Hospital, Auckland, New Zealand; 4 Howard Hughes Medical Institute, Chevy Chase, MD 20815 USA

**Keywords:** Alzheimer’s disease, Unfolded protein response, Olfactory bulb, Anterior olfactory nucleus, PERK, eIF2α

## Abstract

Olfactory dysfunction is an early and prevalent symptom of Alzheimer’s disease (AD) and the olfactory bulb is a nexus of beta-amyloid plaque and tau neurofibrillary tangle (NFT) pathology during early AD progression. To mitigate the accumulation of misfolded proteins, an endoplasmic reticulum stress response called the unfolded protein response (UPR) occurs in the AD hippocampus. However, chronic UPR activation can lead to apoptosis and the upregulation of beta-amyloid and tau production. Therefore, UPR activation in the olfactory system could be one of the first changes in AD. In this study, we investigated whether two proteins that signal UPR activation are expressed in the olfactory system of AD cases with low or high amounts of aggregate pathology. We used immunohistochemistry to label two markers of UPR activation (p-PERK and p-eIF2α) concomitantly with neuronal markers (NeuN and PGP9.5) and pathology markers (beta-amyloid and tau) in the olfactory bulb, piriform cortex, entorhinal cortex and the CA1 region of the hippocampus in AD and normal cases. We show that UPR activation, as indicated by p-PERK and p-eIF2α expression, is significantly increased throughout the olfactory system in AD cases with low (Braak stage III-IV) and high-level (Braak stage V-VI) pathology. We further show that UPR activation occurs in the mitral cells and in the anterior olfactory nucleus of the olfactory bulb where tau and amyloid pathology is abundant. However, UPR activation is not present in neurons when they contain NFTs and only rarely occurs in neurons containing diffuse tau aggregates. We conclude that UPR activation is prevalent in all regions of the olfactory system and support previous findings suggesting that UPR activation likely precedes NFT formation. Our data indicate that chronic UPR activation in the olfactory system might contribute to the olfactory dysfunction that occurs early in the pathogenesis of AD.

## Introduction

Olfactory dysfunction is an early and prevalent symptom of Alzheimer’s disease (AD) that can precede the diagnostic memory and cognitive symptoms by many years [4, 12]. Pathologically, AD is characterised by the accumulation of beta-amyloid plaques and tau neurofibrillary tangles (NFTs), which also accumulate very early in the disease process in the olfactory bulb [23]. Beta-amyloid and NFT aggregates can disturb many cellular processes, such as protein degradation, that can lead to endoplasmic reticulum (ER) stress and activation of the unfolded protein response (UPR). Under normal physiological conditions, activation of the UPR triggers a signalling cascade that restores proteostasis. However, chronic activation of the UPR can initiate apoptosis as has been shown in neurodegenerative diseases such as AD [11, 44].

The UPR consists of three parallel signalling pathways that are mediated by ER membrane proteins: Protein kinase RNA-like ER kinase (PERK), inositol-requiring enzyme 1 (IRE-1), and activating transcription factor 6 (ATF-6). In this study, we focused on the PERK pathway, in which activation has previously been reported in post-mortem AD tissue [18, 20, 43, 47]. In response to an accumulation of misfolded proteins, the ER chaperone BiP/GRP78 is released from PERK and binds to these misfolded proteins. This leads to the dimerisation and autophosphorylation of PERK and subsequent phosphorylation of its downstream effector eukaryotic translation initiator factor 2α (eIF2α). Activated eIF2α inhibits protein synthesis to alleviate the accumulation of misfolded proteins, but increases translation of ATF4 which can drive apoptosis via C/EBP-homologous protein (CHOP). Prolonged activation of this proapoptotic pathway of the UPR leads to synaptic failure and neuronal loss [29].

Several post-mortem tissue studies have demonstrated that UPR activation via the PERK pathway occurs in the human hippocampus, frontal cortex and temporal cortex in AD [18, 20, 43, 47]. These studies further showed that markers of activated UPR such as phosphorylated PERK (p-PERK) and phosphorylated eIF2α (p-eIF2α) are observed in neurons that contain diffuse tau aggregates, but not those with dense NFTs [18, 32]. Therefore, it is hypothesised that UPR activation precedes tau formation in AD and is one of the early mechanisms involved in AD pathogenesis.

**Fig. 1:**
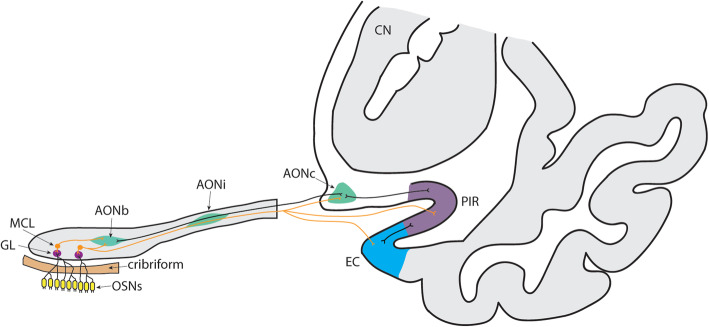
Schematic of the human olfactory pathway. The pathway of primary odour information from the olfactory sensory neurons (OSNs) in the olfactory epithelium of the nose to the anterior olfactory nucleus (AON), piriform cortex (PIR) and entorhinal cortex (EC) via mitral cell projections. This circuitry is based on the olfactory circuitry of the mouse brain and has been extrapolated to the human brain. Abbreviations: AONb, bulbar anterior olfactory nucleus; AONi, intrapeduncular anterior olfactory nucleus; AONc, cortical anterior olfactory nucleus; CN, caudate nucleus; GL, glomerular layer; MCL, mitral cell layer

As olfactory dysfunction is one of the earliest symptoms in AD and tau and beta-amyloid aggregates accumulate in the olfactory bulb very early in the disease, we hypothesised that UPR activation in the olfactory system could be one of the early changes that occur in AD. To provide support for this hypothesis, we used immunohistochemistry to investigate whether the UPR markers p-PERK and p-eIF2α were present or increased in the olfactory system of AD cases with low and high grades of pathology. Our study included the bulbar and intrapeduncular segments of the anterior olfactory nucleus (AON) in the olfactory bulb and downstream cortical olfactory areas including the piriform and entorhinal cortex (Fig. 1), as well as the hippocampal CA1 region. We show that the UPR is activated throughout the olfactory system in all AD cases. We further show that markers of UPR activation infrequently co-occur in the same cell as tau aggregates, and only do so with diffuse tau and not NFTs, as described in previous studies. Our results highlight the concept that while UPR activation may initially be a neuroprotective process, chronic activation could be a mechanism of cellular dysfunction that drives aggregate formation and underlies olfactory dysfunction in early AD.

## Materials and methods

### Human tissue acquisition and processing

Human post-mortem brain tissue was obtained from the Neurological Foundation Human Brain Bank and the Human Anatomy Laboratory within the Department of Anatomy and Medical Imaging, University of Auckland, New Zealand. The tissue was donated with informed consent from the family prior to brain removal and all procedures were approved by the University of Auckland Human Participants Ethics Committee (Ref: 011654). All cases used in this study were assessed by an independent neuropathologist who determined the Braak staging, thal phase and NIA-AA score. The 12 neurologically normal donor cases had no history of neurological abnormalities and no other neuropathology was noted. The mean age (± standard deviation) of the normal cases was 73 ± 12.9 years and ranged from 56 to 94 years. The average post-mortem delay was 25 ± 9.7 h with a range of 13 to 48 h (Table 1). The 16 AD donor cases had a clinical history of dementia and the clinical AD diagnosis was confirmed by an independent pathologist. The average age of AD cases was 79 ± 10 years and ranged from 60 to 94 years. The average post-mortem delay was 8.9 ± 4.8 h with a range of 3.5 to 15 h (Table 1).

**Table 1:** Case information for tissue used in this study

Case	Pathology Diagnosis	Age	Sex	PMD	Braak Stage	Β-amyloid Thal Phase	NIA-AA Score (AD severity)
AZ71	AD	68	F	6	VI	5	A3 B3 C2 (high)
AZ75	AD	94	F	3.5	V	5	A3 B3 C2 (high)
AZ83	AD/LBD (neocortical/diffuse)	60	F	16	VI	5	A3 B3 C2 (high)
AZ84	AD/LBD (neocortical)	82	M	18.5	III	5	A3 B2 C1 (intermediate)
AZ86	AD/LBD (neocortical/diffuse)	92	M	8.5	III	5	A3 B2 C2 (intermediate)
AZ90	AD/LBD (amygdala)	73	M	4	V	5	A3 B3 C2 (high)
AZ95	AD/LBD (amygdala)	69	M	12	V	5	A3 B3 C2 (high)
AZ96	AD/LBD (amygdala)	74	F	8.5	V	5	A3 B3 C2 (high)
AZ99	AD	94	F	8.5	V-VI	5	A3 B3 C2 (high)
AZ101	AD	75	M	12.5	VI	4	A3 B3 C2 (high)
AZ103	AD	87	M	24	IV	4	A3 B2 C2 (intermediate)
AZ104	AD/LBD (neocortical/diffuse)	79	F	13	IV	5	A3 B2 C2 (intermediate)
AZ109	AD	90	F	31	III	4	A3 B2 C1 (intermediate)
AZ113	AD	77	M	3.5	VI	5	A3 B3 C2 (high)
AZ120	AD/LBD (amygdala)	74	F	6.5	IV	5	A3 B2 C2 (intermediate)
H251	AD	77	M	11.5	III	5	A3 B2 C3 (intermediate)
OFB55^a^	NORMAL	56	M	26	–	–	–
OFB57^a^	NORMAL	63	M	36	–	–	–
OFB59^a^	NORMAL	67	F	20	–	–	–
OFB6A^a^	NORMAL	82	M	24	–	–	–
OFB8A^a^	NORMAL	87	M	48	–	–	–
H190	NORMAL	72	F	19	0	–	–
H230	NORMAL	57	F	32	0	–	–
H240	NORMAL	73	F	26.5	I-II	–	–
H242	NORMAL	61	M	19.5	0	–	–
H243	NORMAL	77	M	13	0	–	–
H246	NORMAL	89	F	17	I-II	–	–
H250	NORMAL	94	M	19	I-II	–	–

*Abbreviations*: *LBD* Lewy body disease

^a^only olfactory bulbs were obtained for these cases

The right hemisphere of each brain was fixed by perfusion of 15% formaldehyde in 0.1 M phosphate buffer through the cerebral arteries and the hemisphere was subsequently dissected into approximately 60 blocks as described in previous publications [48]. From each block, a 0.5 cm-thick section was selected for paraffin-embedding and the remaining tissue was snap frozen using dry-ice snow and stored at − 80 °C. The olfactory bulbs were removed from the brain prior to perfusion to preserve tissue integrity and were immersion fixed in 15% formaldehyde. The olfactory bulbs and brain tissue blocks were processed for paraffin embedding as described previously [50]. The paraffin blocks were sectioned at a thickness of 7 μm using a rotary microtome (Leica Biosystems RM2335). The olfactory bulbs were sectioned in the sagittal plane, while the hippocampus was sectioned coronally. The sections were floated on a water bath set at 37 °C (Leica Biosystems, HI1210) before being mounted on Superfrost Plus slides (Menzel- Glaser) and air-dried for 18 h at room temperature.

Piriform cortex tissue was not available on the paraffin-embedded tissue blocks and was instead obtained from the fixed-frozen tissue blocks. These blocks were sectioned at 50 μm on a freezing sliding microtome and stored at 4 °C in phosphate-buffered saline (PBS) containing 0.1% sodium azide.

### Immunofluorescent labelling of paraffin sections

Paraffin sections underwent two sequential rounds of immunofluorescent triple labelling to stain four markers of interest plus either p-PERK or p-eIF2α on each section. For each brain region, three sections were labelled for each staining combination per case. For the olfactory bulb, the most central section of the bulb and two sections approximately 350 μm either side were selected. For the CA1 and entorhinal cortex (EC) analysis, three coronal sections spaced 100 μm apart from the central 2 cm of the hippocampal formation were selected.

The fluorescent triple labeling procedure follows the method described in Stevenson et al., (2020) [42]. For the olfactory bulb and hippocampus sections, the slides were heated at 60 °C for 1 h (h) to melt the paraffin wax before being cleared in xylene (2 × 30 min) and rehydrated in an ethanol series of 100%, 2 × 10 min; 95%, 5 min; 80%, 5 min; and 75% ethanol, 5 min. Heat-induced epitope retrieval was performed using 10 mM sodium citrate buffer with 0.05% Tween 20, pH 6.0 in a pressure cooker (2100 Antigen Retriever, Aptum Biologics Ltd.) for 20 min at 121 °C and left to cool for 1.5 h. The slides were then washed in PBS and permeabilised in PBS + 0.1% Triton X-100 for 15 min at room temperature. To block for non-specific binding of the secondary antibodies, the sections were incubated in 10% normal goat serum (Gibco #16210–072) for 1 h at room temperature. The first round of primary antibodies (Table 2) included PGP9.5 and either p-eIF2α, p-PERK, CK1δ or p-IRE1α diluted in 1% normal goat serum. The sections were incubated in the primary antibody mixture overnight in a humidified chamber at 4 °C. Secondary antibodies - goat anti-rabbit Alexa Fluor 488 (ThermoFisher; A11034) and goat anti-mouse Alexa Fluor 647 (ThermoFisher; A21236) – together with Hoechst 33342 nuclei stain (1: 20,000 dilution, Molecular probes; H1399) were diluted in 1% normal goat serum and applied for 3 h at room temperature. The sections were washed and coverslipped with Prolong® Gold mounting media (Molecular Probes; P36930). After each step, the sections were washed for 3 × 5 min in PBS.

**Table 2:** Primary antibodies used in this study

Antigen	Species	Dilution	Manufacturer	Catalogue Number	RRID
Phospho-eIF2α (pSer^51^)	rabbit	1:500	Millipore-Sigma	SAB4504388	AB_2847826
Phospho-PERK (Thr981)	rabbit	1:500	Santa Cruz	sc-32,577	AB_2293243
Phospho-IRE1α (pSer^724^)	rabbit	1:500	Novus Biologicals	NB100–2323	AB_10145203
CK1δ	rabbit	1:400	ThermoFisher	PA5–32129	AB_2549602
PGP9.5	mouse	1:500	Abcam	Ab8189	AB_306343
NeuN	guinea pig	1:1000	Millipore-Sigma	ABN90P	AB_2341095
Tau	rabbit	1:2000	Aligent	A0024	AB_10013724
Beta-amyloid	mouse	1:100	Aligent	M0872	AB_2056966

Sections were imaged using an automated fluorescence microscope (Zeiss Z2 Axioimager) equipped with MetaSystems VSlide slide scanner (MetaSystems) running MetaFer (V 3.12.1) with a 20x air objective (0.9 NA). Following imaging, the sections were immersed in PBS and carefully decoverslipped for the second round of staining. The sections were immersed in 80% formic acid for 5 min, which served to eliminate the previous fluorescent staining and retrieve the tau and beta-amyloid aggregate epitopes. The washing, permeabilisation and blocking steps were repeated as described above and the second round of primary antibodies (NeuN, Tau, and Amyloid) were added to the sections overnight at 4 °C. The secondary antibodies (goat anti-rabbit Alexa Fluor 488 (ThermoFisher A11034), goat anti-mouse Alexa Fluor 594 (ThermoFisher A11032) and goat anti-guinea pig 647 (ThermoFisher A21450) were diluted in 1% normal goat serum and Hoechst 33342 nuclear stain was added. Secondary antibodies were added for 3 h at room temperature, after which the sections were washed, coverslipped with Prolong Gold AntiFade mounting media and imaged again.

### Immunofluorescent labelling of free-floating piriform cortex sections

As the piriform cortex tissue was not available on paraffin-embedded tissue blocks, it was processed from the fixed-frozen tissue blocks. Three sections spaced 200 μm apart were selected for each staining combination. The human piriform cortex traverses the junction of the temporal and frontal lobes and in this study, we used the temporal aspect of the piriform cortex located anterior to the amygdala which shows distinct three-layered cytoarchitecture. These sections were labelled using free-floating fluorescent immunohistochemistry as previously described [31]. The two sequential rounds of fluorescent staining could not be performed on free-floating sections so the co-labelling of p-PERK and p-eIF2α with tau was not investigated in this region. Sequential sections were stained for p-PERK and NeuN, p-eIF2α and NeuN, and tau, amyloid and NeuN. The sections labelled for p-PERK and p-eIF2α underwent heat-induced epitope retrieval using 10 mM sodium citrate buffer with 0.05% Tween 20, pH 6.0 and rapid heating to 100 °C using a microwave for 30 s, followed by cooling at room temperature for 1 h. The sections labelled for tau and beta-amyloid underwent antigen retrieval using 80% formic acid for 5 min at room temperature. All sections were blocked for non-specific secondary antibody binding by incubating in 10% normal goat serum (Gibco #16210–072) for 1 h at room temperature. Primary antibodies (Table 2) were diluted in 1% normal goat serum and sections were incubated in this solution for 48 h at 4 °C. Subsequently, the relevant species-specific secondary antibodies and nuclear counterstain (as above) were applied for 24 h at room temperature. Between steps, the sections were washed in PBS with 0.1% Triton X-100. Finally, the sections were mounted onto slides and coverslipped using Prolong Gold AntiFade mounting media (Molecular Probes #P36930). Imaging was carried out as for the paraffin sections.

### Alignment and segmentation of images

The fluorescent images from the paraffin sections were separated into the individual channels as 8-bit greyscale images. The Hoechst labelling was present for every staining and imaging round so nuclei were used as the intrinsic markers for image registration between staining rounds. We used a custom-designed registration code in Python to automatically register the nuclei images. We found that the accuracy of the registration was improved when the images were pre-processed before the registration. We applied a 50-pixel rolling background subtraction and a 5-pixel median filter to the nuclei images to smooth out any nuclei staining fluctuations. The processed nuclear images were registered with each other using a Jupyter Notebook to implement an AKAZE affine registration and a transformation matrix was extracted for each image set [2]. Subsequently, we applied this transformation matrix to all the individual images for the set. The individual images were merged together into a single file and pseudo-coloured using ImageJ v1.52p.

### Semi-quantitative analysis of p-eIF2α, p-PERK, tau and NeuN+ cells

**Fig. 2:**
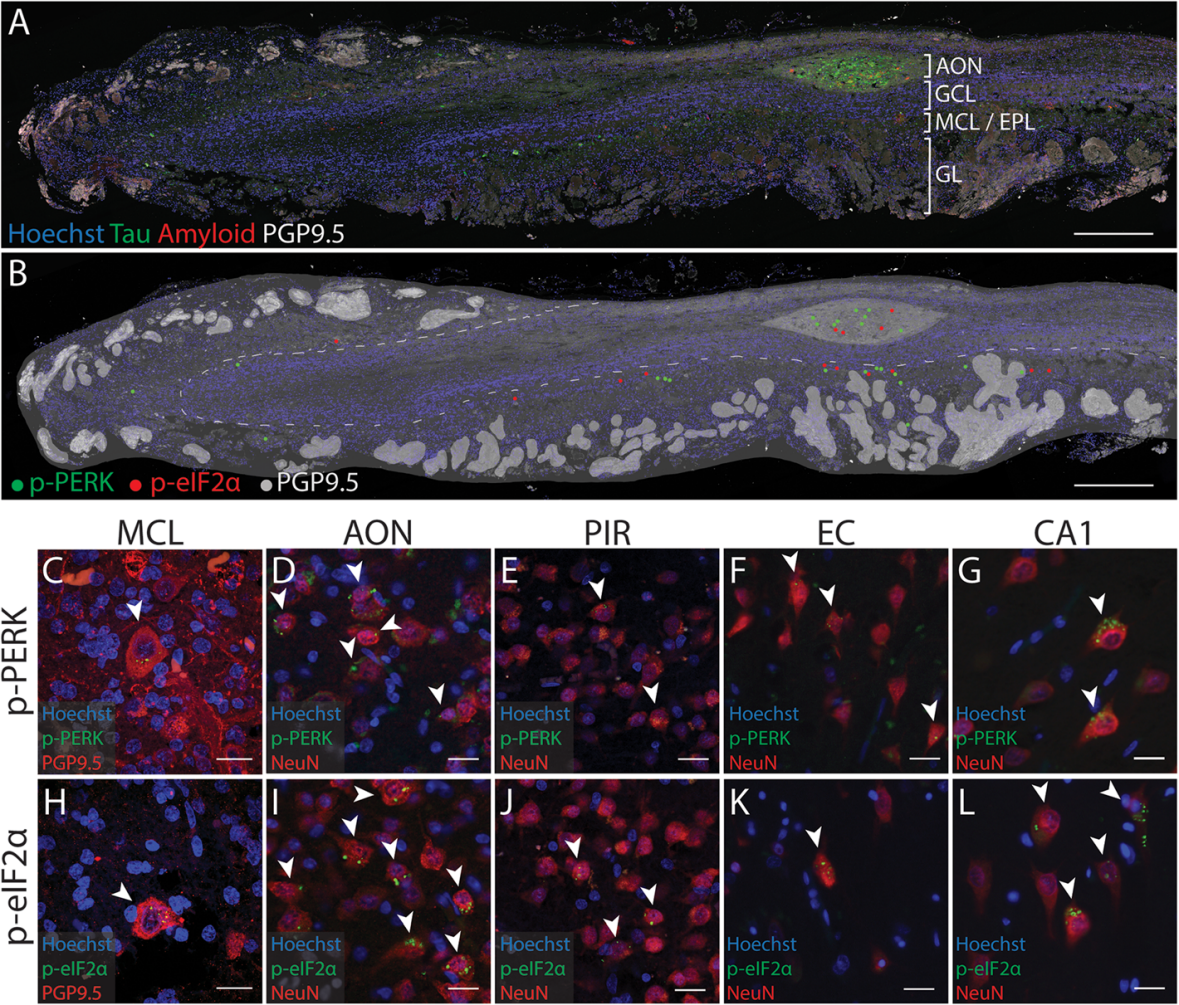
Distribution of p-PERK and p-eIF2α in the human AD olfactory bulb relative to tau and beta-amyloid aggregates. **a** Representative sagittal section of a human AD olfactory bulb labelled for tau, beta-amyloid, and PGP9.5 with Hoechst (nuclei) stain, indicating the location of aggregates within the anterior olfactory nucleus (AON) and the mitral cell layer/ external plexiform layer (MCL/EPL). **b** A schematic diagram of the location of markers overlaid on the same sagittal section. The outline of the olfactory bulb is drawn in dark grey. The glomeruli and the AON have been traced in light grey based on PGP9.5 immunoreactivity. The locations of p-PERK+ cells are indicated by green dots and the locations of p-eIF2α + cells on an adjacent section have been indicated by red dots. p-PERK+ and p-eIF2α + cells were predominantly located in the AON and MCL/EPL. Neurons containing p-PERK (C-G, arrows) and p-eIF2α (H-L, arrows) were identified in all the olfactory regions investigated in this study. Abbreviations: CA1, Cornu Ammonis 1; EC, entorhinal cortex; GCL, granule cell layer; GL, glomerular layer; PIR, piriform cortex. Scale bars = 500 μm (A-B), 20 μm (C-L)

All measurements and cell counts were performed using ImageJ v1.52p. The area of the region of interest (ROI) was first measured using the polygon selection tool on the merged image. The olfactory bulb AON is subdivided into a series of compartments throughout the olfactory bulb and tract. These compartments can be delineated based on the high PGP9.5 immunoreactivity and an abundance of diffuse neuronal nuclei which are evident with Hoechst staining (Fig. 2a-b). The AON compartments are grouped based on their location: the bulbar AON (AONb) encompasses all compartments within the bulb, while the intrapeduncular AON (AONi) encompasses all compartments within the olfactory tract [37]. The piriform cortex, entorhinal cortex and CA1 region of the hippocampus were delineated according to the Allen Human Brain Atlas [3, 13].

Once the ROI was delineated, for each image channel the background fluorescence intensity was measured from a 15 μm^2^ area of the ROI in triplicate and averaged. Starting with the p-eIF2α or p-PERK channel, cells with positive staining were selected using the multipoint region tool in ImageJ. One multipoint marker was placed on the brightest p-eIF2α or p-PERK spot in the cell. For all remaining cells, those that were tau+ and NeuN+ were selected by placing a multipoint marker. As all three antibody labels are cytoplasmic, this method resulted in each cell within the image containing one multipoint marker at the same coordinate for all three image channels. At each marker coordinate the point intensity was measured for each image channel as grey values between 0 and 255 and subtracted from the background average for that channel. To be considered a positively stained cell, the point intensity needed to be above a determined threshold for that channel (grey values above background: 35 for tau, 15 for NeuN and 30 for p-eIF2α or p-PERK). Therefore, if a cell was positive for more than one marker then it would be indicated by point intensities above threshold for each image channel at that marker coordinate. A subset of the images were counted by a second investigator to ensure the counting method was repeatable. The data are presented as the percentage of neurons (NeuN+ cells) containing p-eIF2α or p-PERK or the number of p-eIF2α or p-PERK cells per mm^2^. To investigate the amount of co-labelling between tau and p-eIF2α or p-PERK we have also presented the data as the percentage of tau+ cells that also contain p-eIF2α or p-PERK and the percentage of p-eIF2α or p-PERK+ cells that also contained tau. We investigated the correlation between the percentage of p-PERK+ and p-eIF2α + neurons and the age or post-mortem delay for each case using a Spearman’s correlation coefficient. There was no significant correlation with age in any of the regions assessed (Additional file 1: Supplementary Figure 1 A – E). There was no significant correlation with post-mortem delay in the CA1 regions. However, there were moderate inverse correlations with post-mortem delay in other regions (Additional file 1: Supplementary Figure 1 F – J). This is likely due to the normal cases used in this study having longer post-mortem delays than the AD cases. To control for this difference, we investigated the correlation for cases that had a post-mortem delay between 11.5 and 36 h as this range overlapped between the two groups. There was no significant correlation between the percentage of p-eIF2α + or p-PERK+ neurons and post-mortem delay for these cases, indicating that our results are unlikely to reflect a loss of phospho-epitopes in control cases due to post-mortem delay (Additional file 1: Supplementary Figure 1K – O).

### Semi-quantitative analysis of tau and beta-amyloid load

To measure the total load of tau and beta-amyloid, the area of the ROI was first measured using the polygon selection tool. The threshold tool was then used to determine the area of the ROI covered by tau or beta-amyloid immunoreactivity. A threshold of at least 40 grey values was used to detect true labelling above background. The thresholded area was normalised to the total ROI area to obtain the percentage area of aggregate labelling.

### Statistical analysis

Data visualisation and statistical analysis were performed using GraphPad Prism Version 8.03. All data are presented as mean ± standard deviation (SD) from the total values across three different sections per case. To test for differences between AD and normal cases, a parametric unpaired t-test was used for regions where the data fit the assumptions of normal distribution and equality of variance. If the data did not satisfy these assumptions then a non-parametric Mann-Whitney test was performed. To test for differences in the percentage of neurons containing p-eIF2α or p-PERK across Braak stages, a non-parametric Kruskal-Wallis test with Dunns multiple comparisons test was performed for each region, as not all groups fit the assumptions for parametric testing. To investigate the statistical significance of correlations between the percentage of neurons containing p-eIF2α or p-PERK and tau or beta-amyloid load, a Spearman’s correlation coefficient was used. Statistical significance was set as *p* < 0.05.

## Results

### p-PERK and p-eIF2α are located in areas of the olfactory bulb affected by tau and beta-amyloid aggregation in AD

To study UPR activation in the olfactory system of AD patients and normal individuals, we performed immunohistochemistry with p-PERK and p-eIF2α antibodies on post-mortem human brain sections. We used antibodies that have been used in previous studies of human brain tissue [17, 18, 32] and co-labelled with antibodies for tau and beta-amyloid on the same section to determine co-localisation. For consistency, we used the same tau and beta-amyloid antibodies that are used by our clinical pathologist for Braak staging in our comparison of olfactory regions to the other brain regions. The tau antibody labels amino acid 243–441 at the C-terminal of human tau protein, independently of phosphorylation sites and the beta-amyloid antibody epitope corresponds to amino acids 8–17.

Tau and beta-amyloid aggregates were detected in olfactory bulbs from AD cases. These aggregates were most abundant within the AON but also scattered throughout the external plexiform layer and mitral cell layer (Fig. 2a). As it is difficult to delineate the external plexiform layer from the mitral cell layer in the human bulb they have been labelled together in Fig. 2a and b. We also observed that cells expressing p-PERK and p-eIF2α were predominantly located in the AON. These p-PERK+ and p-eIF2α + cells did not appear to be clustered around beta-amyloid plaques in the AON (Additional file 2: Supplementary Figure 2). Furthermore, p-PERK and p-eIF2α immunoreactivity was identified within large neurons (approx 20 μm diameter) with large diffuse nuclei and located along the border of the granule cell layer or within the external plexiform layer (Fig. 2b, c, h). The size and location of these cells are consistent with previous descriptions of mitral cells [5, 24, 28, 41]. To determine whether the IRE1α branch of the UPR pathway was also activated in the olfactory bulb we labelled for p-IRE1α. In AD cases only, we found very few p-IRE1α + neurons within the AON (Additional File 2: Supplementary Figure 3). We did not observe any p-IRE1α + mitral cells.

### Increase in p-PERK+ and p-eIF2α + cells throughout the human olfactory system in AD compared to neurologically normal cases

We investigated whether UPR activation was present in downstream olfactory regions in AD and normal cases. We found p-PERK and p-eIF2α immunoreactivity as punctate granular staining in the cell cytoplasm resembled granulovacuolar degeneration, as described in other studies [18, 32, 40, 43, 49]. Labelling for the granulovacuolar degeneration marker CK1δ was also observed in these regions of the olfactory bulb and hippocampus (Additional file 2: Supplementary Figure 3) In all regions we assessed, nearly all cells with p-PERK and p-eIF2α immunoreactivity also co-labelled with the neuronal marker NeuN and were therefore considered to be p-PERK+ neurons or p-eIF2α + neurons. We observed p-PERK+ neurons (Fig. 2c – G) and p-eIF2α + neurons (Fig. 2h – l) in all the olfactory and hippocampal regions in AD cases.

**Fig. 3:**
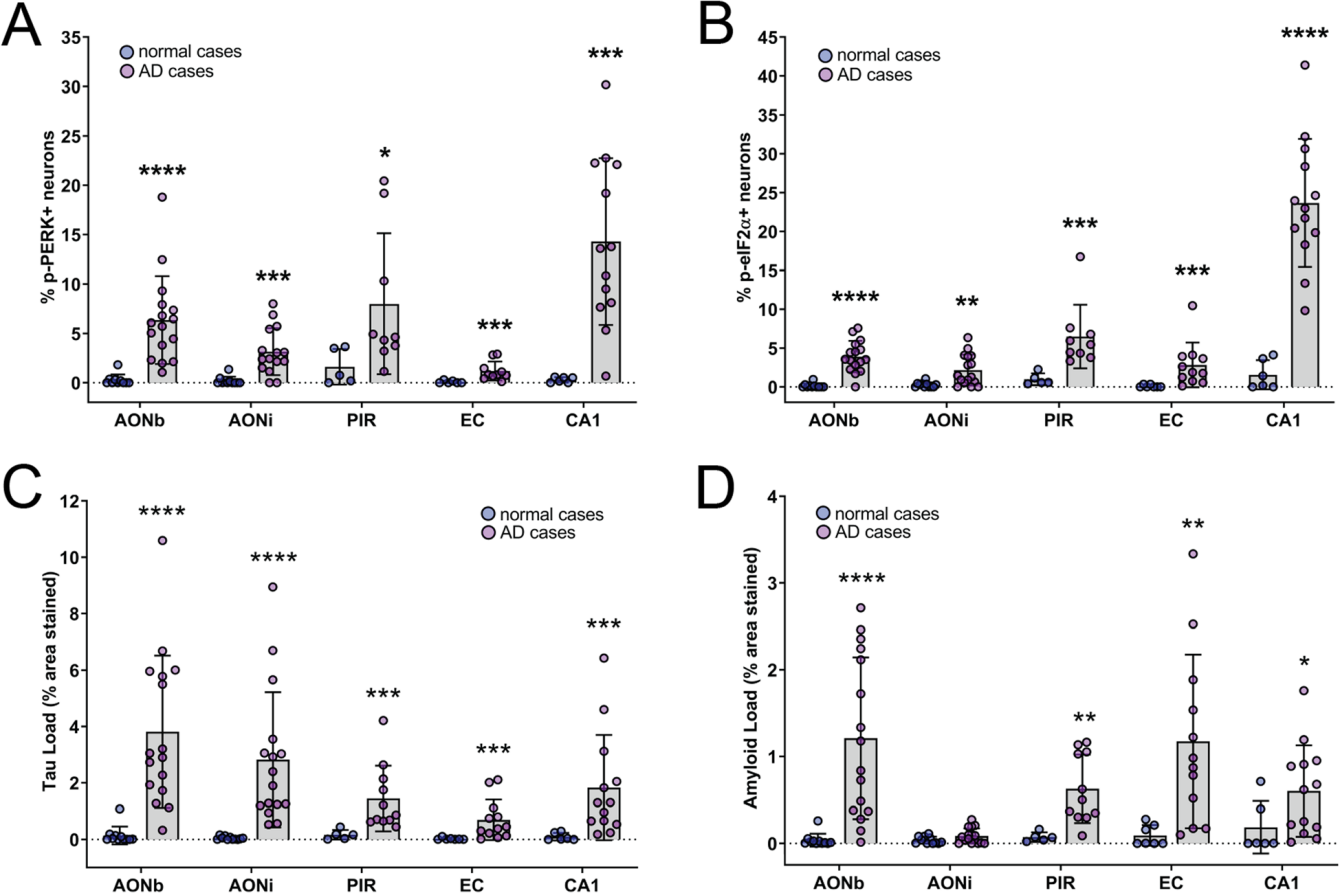
Quantification of p-PERK+ and p-eIF2α + neurons, tau load and beta-amyloid load in regions of the human olfactory system in normal and AD cases. The percentage of p-PERK+ neurons (**a**) and p-eIF2α + neurons (**b**) was determined in the AONb, AONi, piriform cortex, entorhinal cortex and CA1 region of AD and normal cases. There was a significant increase in both p-PERK and p-eIF2α for AD cases in all regions assessed. Tau load (**c**) and beta-amyloid load (**d**) for each region was determined as the % area of the image stained. Tau load was significantly increased in AD cases for all regions assessed. Similarly, the beta-amyloid load was significantly increased in AD cases for all regions except the AONi. *P ≤ 0.05, **P ≤ 0.01, ***P ≤ 0.001, ****P ≤ 0.0001 compared to normals in the same region

We subsequently quantified the number of neurons and total cells expressing p-PERK and p-eIF2α. The percentage of p-PERK+ neurons was significantly increased in AD cases for all the olfactory and hippocampal regions assessed in this study (Fig. 3a, Additional file 3: Supplementary Table 1). The analysis of p-PERK cell density showed that the number of p-PERK+ cells per mm^2^ was also significantly increased in AD cases compared to normals for all regions except the piriform cortex (Additional file 4: Supplementary Figure 5). Within AD cases, there was no significant difference in the amount of p-PERK between the olfactory regions (AONb, AONi, piriform cortex) and all regions except the AONi had significantly more p-PERK+ neurons than the entorhinal cortex (Additional file 3: Supplementary Table 2).

The quantification of p-eIF2α immunoreactivity showed a significant increase in the percentage of p-eIF2α + neurons for AD cases for all regions (Fig. 3b, Additional file 3: Supplementary Table 1). The number of p-eIF2α + cells per mm^2^ was also significantly increased in AD cases compared to normals for all regions (Additional file 4: Supplementary Figure 5). Similar to p-PERK, there was no significant difference in the amount of p-eIF2α across the olfactory regions within AD cases. However, the CA1 region had significantly more p-eIF2α + neurons than the AONb, AONi and EC ((Additional file 3: Supplementary Table 2).

This quantification showed little to no p-PERK+ or p-eIF2α + neurons in normal cases. Taken together, these data indicate that UPR activation is substantially increased throughout the olfactory and hippocampal regions of AD cases. The amount of activation is also relatively consistent throughout the AONb, AONi and piriform cortex.

### Tau and beta-amyloid load is increased in the human olfactory system in AD compared to neurologically normal cases

We next evaluated whether tau and beta-amyloid load were increased in the regions where UPR activation was identified. We found that tau load was significantly increased in all the regions we assessed in AD cases compared to normals (Fig. 3c, Additional file 3: Supplementary Table 3). Similarly, the beta-amyloid load was significantly increased in all regions, except the AONi in AD cases compared to normals (Fig. 3d, Additional file 3: Supplementary Table 3). Within AD cases, tau load in the AONb and AONi was significantly higher than that of the entorhinal cortex, and beta-amyloid load in the AONi was significantly lower than all other regions (Additional file 3: Supplementary Table 4). Together with the p-PERK and p-eIF2α labelling, these data indicate that tau and beta-amyloid pathology are significantly increased in the olfactory regions of AD cases where UPR activation also occurs.

### Neurons with p-PERK and p-eIF2α are found in olfactory regions of AD brains with low-grade NFT pathology

**Fig. 4:**
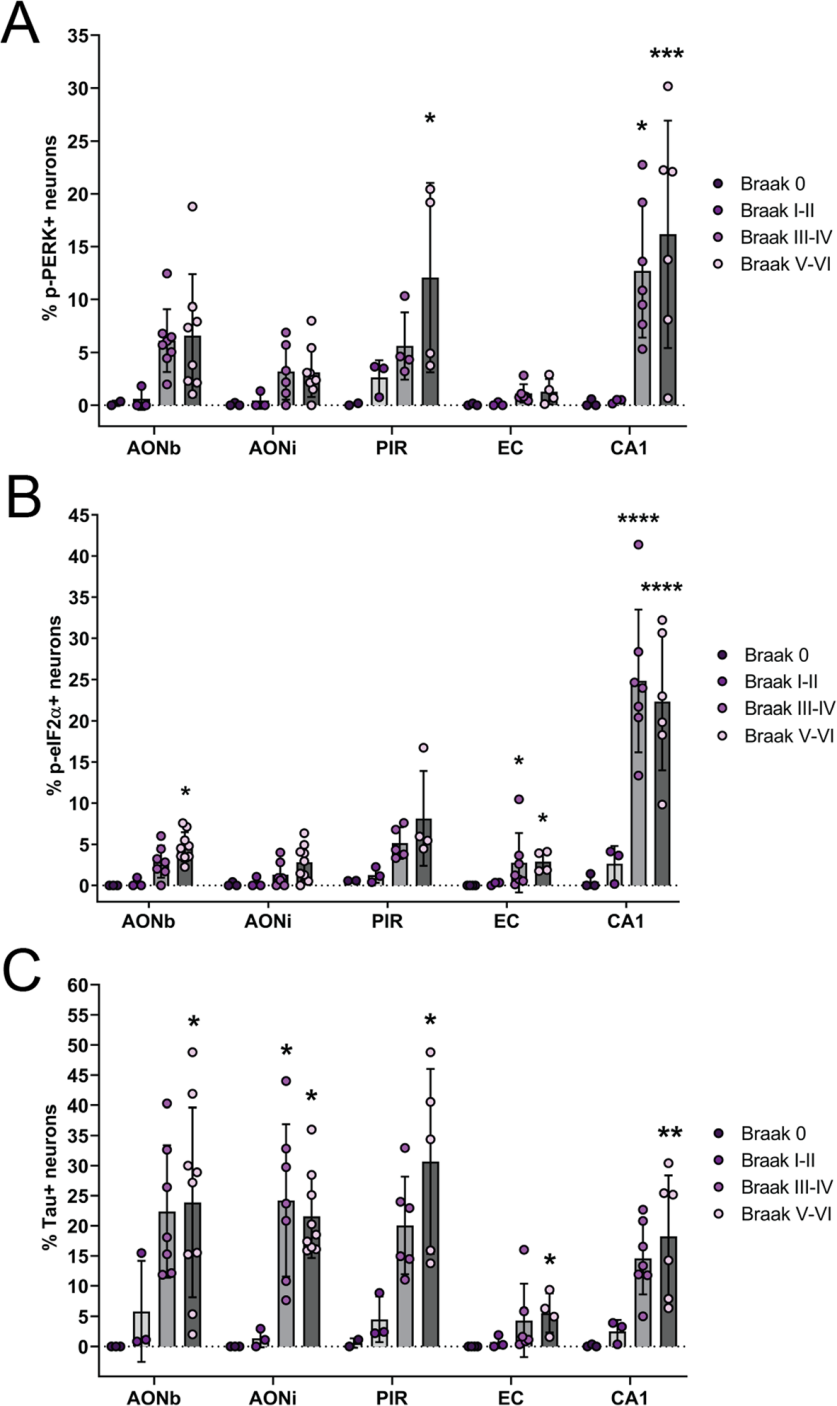
Quantification of p-PERK+, p-eIF2α+ and tau+ neurons in each region at different stages of pathology. Cases were grouped based on their Braak stage, with stage 0 and stage I-II indicating normal cases with no or very minimal tau pathology respectively, stage III-VI indicating AD cases with mild, low-level tau pathology and stage V-VI indicating AD cases with advanced, high-level tau pathology. Across all regions assessed, the percentage of p-PERK+ neurons (**a**) and p-eIF2α + neurons (**b**) was increased in cases with Braak stage III-IV and V-VI pathology compared to Braak stage 0 or I-II. This increase was statistically significant only in the piriform cortex and CA1 region for p-PERK and in the AONb, entorhinal cortex and CA1 region for p-eIF2α. There was no significant difference in p-PERK+ or p-eIF2α + neurons in cases with low Braak stage AD pathology (III-IV) compared to high Braak stage AD pathology (V-VI) in any region. (**c**) Similar results were observed for the percentage of tau+ neurons. *P ≤ 0.05, **P ≤ 0.01, ****P ≤ 0.0001 compared to Braak stage 0 in the same region

To investigate whether UPR activation was related to the disease stage, we grouped the data according to the Braak stage for each case. We found that the percentage of p-PERK+ neurons (Fig. 4a) and p-eIF2α + neurons (Fig. 4b) were increased in cases with Braak stage III-IV and V-VI pathology compared with Braak stage 0 or I-II. This increase was statistically significant in the piriform cortex and CA1 region for p-PERK and in the AONb, entorhinal cortex and CA1 region for p-eIF2α. There was no significant difference in the percentage of p-PERK+ or p-eIF2α + neurons between cases with Braak stage III-IV and Braak stage V-VI in any region (Additional file 3: Supplementary Table 5).

A similar result was observed for the percentage of tau+ neurons, which was increased in cases with Braak stage III-IV and V-VI pathology compared to Braak stage 0 or I-II (Fig. 4c). This increase was statistically significant in the AONb, AONi, piriform cortex, and CA1 region. There was no significant difference in the percentage of tau+ neurons between cases with Braak stage III-IV and Braak stage V-VI in any region (Additional file 3: Supplementary Table 5).

### p-PERK+ and p- eIF2α + neurons contain diffuse tau aggregates rather than NFTs

**Fig. 5:**
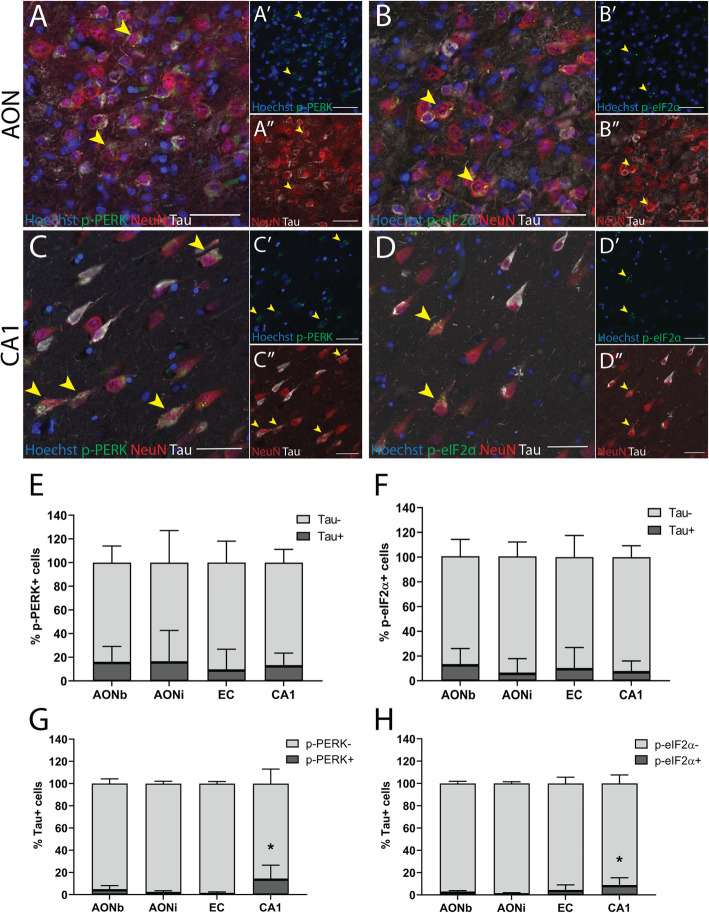
Co-labelling of p-PERK and p-eIF2α with tau in AD cases. (**a-d**) Fluorescent triple labelling of p-PERK and p-eIF2α with NeuN and tau in the AON (**a**-**b**) and CA1 region (**c**-**d**). NeuN+ cells that contained p-PERK or p-eIF2α and tau (arrowheads) often had diffuse tau aggregates rather than dense neurofibrillary tangles. (**e**-**f**) Stacked bar graph indicating the percentages of p-PERK+ cells (**e**) or p-eIF2α + cells (fα) that did or did not contain tau. A low percentage of p-PERK+ or p-eIF2α + cells contained tau and this was not significantly different across the olfactory or hippocampal sub-regions we assessed. (**g**-**h**) Stacked bar graph indicating the percentages of tau+ cells that did or did not contain p-PERK (**g**) or p-eIF2α (**h**). A very low percentage of tau+ cells contained p-PERK or p-eIF2α and this was significantly higher in the CA1 region compared to the AONi and EC for p-PERK, and the AONi alone for p-eIF2α. Scale bars = 50 μm

Previous studies have shown UPR activation is limited to neurons that contain diffuse tau rather than dense NFTs and we sought to determine if this was the case for the olfactory regions. In all the regions assessed, NeuN+ cells that contained both tau and p-PERK or p-eIF2α contained diffuse tau aggregates rather than dense NFTs (Fig. 5a - d). The percentage of p-PERK+ cells (Fig. 5e) or p-eIF2α + cells (Fig. 5f) that contained tau was relatively low and was not significantly different across the regions assessed (Additional file 3: Supplementary Table 6 and 7).

The co-labelling of tau and UPR markers was also examined as the percentage of total tau+ cells that contained either p-PERK or p-eIF2α. A very low proportion of the tau+ cells contained p-PERK (Fig. 5g) or *p*-eIF2α (Fig. 5h). Across the regions, the percentage of total tau+ cells that contained either *p*-PERK or p-eIF2α was significantly higher in the CA1 region compared to the AONi and EC for *p*-PERK, and the AONi alone for p-eIF2α (Additional file 3: Supplementary Table 6 and 7). Together, these quantitative data indicate that UPR activation is infrequently accompanied by tau deposition.

### The amount of p-PERK and p-eIF2α in olfactory regions is not associated with the amount of tau or beta-amyloid

**Fig. 6:**
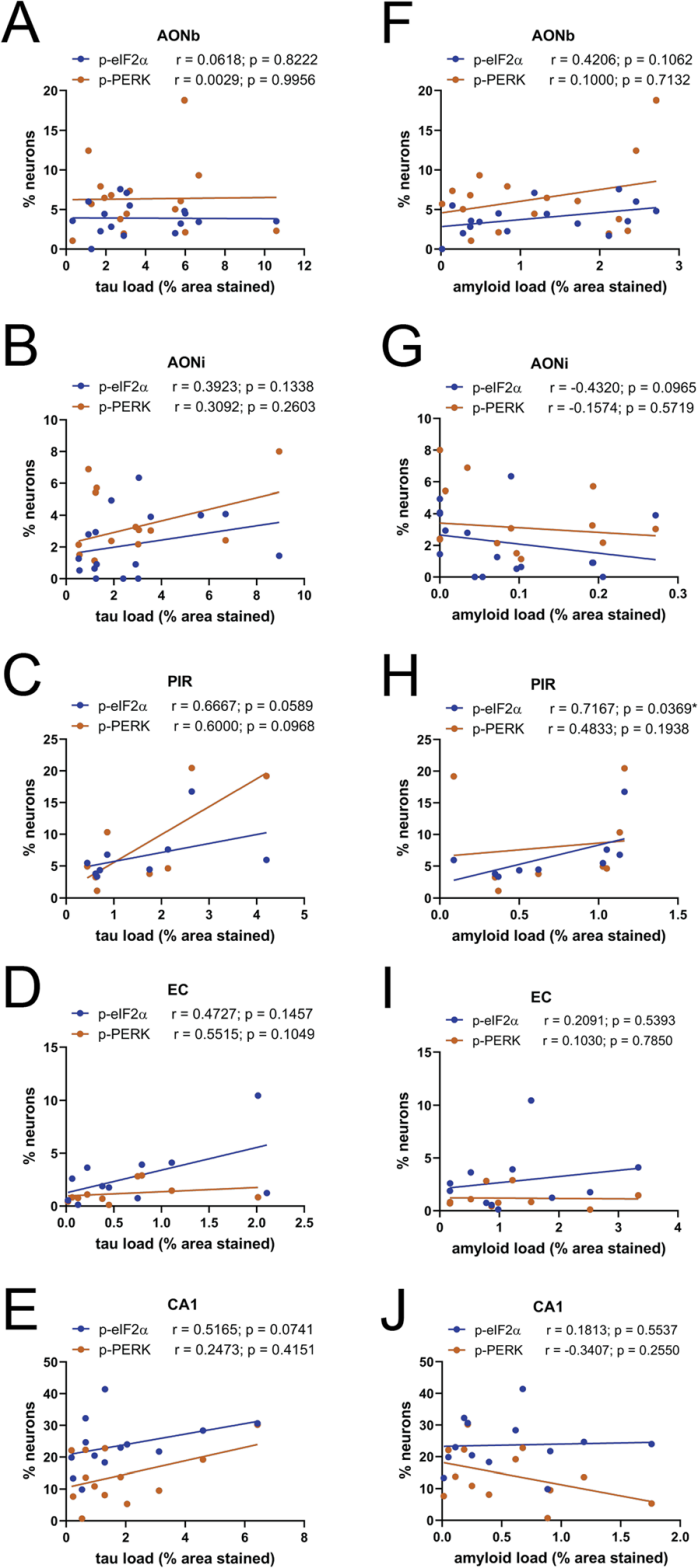
Correlation between percentage of p-PERK+ and p-eIF2α + neurons and tau load or amyloid load in AD cases. For each region, the percentage of p-PERK+ or p-eIF2α + neurons was plotted against tau load (**a**-**e**) and beta-amyloid load (**f**-**j**) for each case. A non-parametric Spearman’s correlation test determined that there was no significant correlation between the percentage of p-PERK+ or p-eIF2α + neurons and tau load in any of the regions assessed. Similarly, there was no significant correlation between the percentage of p-PERK+ or p-eIF2α + neurons and amyloid load in the AONb, AONi, EC and CA1. However, there was a significant moderate positive correlation between the percentage of p-eIF2α + neurons and amyloid load in the piriform cortex

We further investigated whether there is a correlation between tau or beta-amyloid load and the amount of UPR activation in AD cases. There was no significant correlation between the percentage of p-PERK+ or p-eIF2α + neurons and tau load in any of the regions assessed (Fig. 6a – e). Furthermore there was no signficant correlation between percentage of p-PERK+ or p-eIF2α + neurons and the percentage of tau+ neurons in any region, other than the entorhinal cortex where there was a significant positive correlation between the percentage of p-eIF2α + neurons and tau+ neurons (Additional file 4: Supplementary Figure 4). There was also no significant correlation between the percentage of p-PERK+ or p-eIF2α + neurons and beta-amyloid load in the AONb, AONi, EC, and CA1 (Fig. 6f – j). However, there was a significant moderate positive correlation between the percentage of p-eIF2α + neurons and beta-amyloid load in the piriform cortex (Fig. 6h). Overall it was determined that for AD cases there was no consistent relationship between aggregate load and UPR activation in the regions assessed.

## Discussion

In this study, we provide the first evidence that the UPR is activated throughout the human olfactory system in AD. As olfactory deficits are an early and prevalent symptom of AD and pathological tau and beta-amyloid aggregates accumulate in the olfactory bulb very early in the disease progression, our data implicate the UPR as a potential mechanism contributing to the first pathological changes in AD. It is important to acknowledge that like all post-mortem studies, we have reported findings at the end-stage of the disease and our conclusions on the temporal relationship between UPR activation and AD progression are based on the assumption that early or pre-symptomatic AD is reflected by lower stages of brain pathology as indicated by Braak staging.

In the olfactory bulb, we observed that markers of UPR activation p-PERK and p-eIF2α were predominantly clustered in the AON and mitral cells. The AON is easily delineated as a cluster of large nuclei within a distinct region of PGP9.5 immunoreactivity [42]. The mitral cell layer is more difficult to delineate due to the irregular layer structure of the human bulb, the discontinuity of the mitral cell layer and absence of markers to distinguish mitral from tufted cells in human tissue [5, 21, 27]. However, we identified p-PERK and p-eIF2α immunoreactivity in cells that matched the regional and morphological description of mitral cells [5, 24, 28, 41]. These regions where the UPR activation was clustered is also where tau and amyloid aggregates accumulate in the AD bulb. The specificity of this distribution suggests that UPR activation and pathological aggregate deposition are linked, with the AON neurons and mitral cells being particularly sensitive to this form of ER stress and aggregate production.

The AON is a structure of particular interest in neurodegenerative diseases such as AD, Parkinson’s disease (PD), dementia with Lewy bodies, amyotrophic lateral sclerosis (ALS) and frontotemporal dementia (FTD). Olfactory deficits are a feature of all these diseases and protein aggregates including tau, beta-amyloid, alpha-synuclein and TDP43 specifically accumulate in the AON of the olfactory bulb [30, 36, 45, 46]. The reason for this specificity of aggregate accumulation is unknown, but it suggests the AON might be particularly vulnerable to dysfunction and that it might underlie olfactory symptoms. Indeed, aggregate accumulation in the AON is postulated to occur in the same pre-symptomatic window that the olfactory deficits arise in AD and PD [7, 8, 23]. There is also evidence that AON cell loss occurs in AD and PD; however, these studies should be repeated using modern volumetric stereological counting techniques [14, 24, 34]. The degree of cell loss is correlated with the severity of olfactory deficits in PD and while this has not been investigated in AD, it indicates that AON neurodegeneration could be a physical underpinning of olfactory dysfunction [34]. Our results show tau and beta-amyloid load in the AON is similar between AD cases with Braak stage III-IV and Braak stage V-VI pathology. This adds support to previous evidence showing aggregate deposition in the AON early in AD progression.

The specificity of UPR activation in regions affected by disease aggregates is corroborated in other post-mortem studies. For example, UPR activation was identified in the brainstem of progressive supranuclear palsy (PSP) cases, but not AD cases. It was also identified in the hippocampus and frontal cortex of AD cases, but not PSP cases [43]. Similarly in PD, UPR activation has been demonstrated in the substantia nigra where alpha-synuclein aggregates accumulate [17]. Our data similarly show that the UPR is activated in olfactory areas of the AD brain that are also affected by tau and amyloid aggregation. However, while the olfactory and hippocampal areas we studied show similar tau and amyloid load in AD cases, significantly more CA1 neurons contained p-eIF2α than AON neurons. This suggests that additional cell stress pathways may contribute to UPR activation in CA1 neurons. CA1 neurons are particularly vulnerable to energy deprivation, which is a stress factor that can induce eIF2α phosphorylation [33, 38]. This regional vulnerability to UPR activation may contribute to the pattern of cell loss and degeneration observed in AD.

When we assessed p-PERK and p-eIF2α across the olfactory regions in AD cases with different Braak stages of tau pathology, we found low grade (stage III-IV) AD cases had equivalent amounts of UPR activation to high grade (stage V-VI) AD cases. Braak staging is an indication of disease severity based on the assumption of a conserved pattern of progressive aggregate deposition and atrophy across the brain [6]. As Braak stage III and IV indicate cases where the neocortex is mostly spared from aggregate pathology, our results indicate that olfactory regions are affected by aggregates very early in the disease progression. Our identification of UPR activation in low Braak stage cases agrees with previous evidence of UPR activation in the AD hippocampus of low Braak stage cases and supports the idea that UPR activation occurs early in the disease progression [18]. While initial activation of the UPR may have a neuroprotective role to restore proteostasis in response to misfolded protein accumulation, evidence suggests that prolonged activation could contribute to neurodegeneration [19]. This sustained activation of the UPR produces a chronic inhibition of protein translation by p-eIF2α that leads to reduced production of synaptic proteins and a subsequent loss of synapses [29, 39, 40]. In addition, p-eIF2α increases the translation of specific mRNAs, such as BACE1 which is involved in the production of beta-amyloid and ATF4 which induces the expression of genes related to ER chaperone production, autophagy and apoptosis [16]. PERK activation has also been linked to tau phosphorylation by increased activity of GSK-3β, and p-PERK colocalisation with GSK-3β is observed in neurons in AD [10, 18, 26, 35]. It remains to be investigated whether chronic UPR activation leads to synaptic dysfunction in OB regions. Therefore, identifying substantial UPR activation in olfactory regions of cases with low Braak stage pathology suggests a role for this cellular stress response in both the neurodegenerative process and the formation of pathological aggregates in AD.

Many aspects of the relationship between the UPR and AD aggregate pathology remain unclear. While the evidence above indicates that the production of tau and beta-amyloid pathology can be a down-stream consequence of UPR activation, tau may also play an up-stream role in UPR activation. A study by Wiersma et al., 2019, used in-vitro and and in-vivo tau seeding models to show that the development of tau pathology induces the formation of granulovacuolar degeneration bodies (GVBs) which contain p-PERK and p-eIF2α, similar to what is seen in the human AD hippocampus [49]. They show a strong positive correlation between the amount of tau pathology developed as a consequence of increased tau seed treatment and the amount of GVBs that developed, therefore providing strong evidence that GVBs containing UPR activation markers develop as a consequence of intracellular tau pathology. Similarly, a recent report shows that a 35 kDa C-terminal fragment of tau containing all four microtubule-binding repeats can induce UPR activation through PERK in Chinese hamster ovary cells, unlike the full-length 4R tau isoform [15]. There are also reports that oligomeric beta-amyloid is able to activate the UPR in-vitro [9, 25]. In contrast, our data indicate that co-expression of activated UPR markers and tau within the same cell is low, and when it was observed, those cells contained diffuse tau immunoreactivity rather than dense NFTs. This observation was consistent across all the olfactory and hippocampal regions we assessed and is in agreement with previous studies [18, 32, 43]. The low incidence of co-expression specifically with diffuse tau suggests that UPR activation precedes tau pathology, particularly the development of diffuse tau aggregrates into NFTs. It also implies that UPR must be no longer be activated in cells with dense NFTs or that it was never activated in cells with NFTs [43]. Furthermore, our quantitative assessment did not find significant correlations between UPR activation and tau or beta-amyloid load in the olfactory regions of AD cases, indicating that cases with higher aggregate loads did not necessarily have more UPR activation. In accordance with current hypotheses, this result could be interpreted in two ways. Firstly, that UPR activation occurs early in the development of tau and beta-amyloid pathology and declines or plateaus as aggregate loads increase or secondly, that soluble tau load contributes to UPR activation in the AD olfactory system rather than aggregated tau. There is indeed evidence that soluble tau may indirectly activate the UPR through cytoplasmic processes that lead to inhibition of protein degradation and therefore the accumulation of misfolded proteins [1, 40, 43]. These interpretations, in line with current literature, support the conclusion that UPR activation occurs in the earliest stages of tau and beta-amyloid pathology, but it remains unclear whether tau aggregates activate the UPR directly.

UPR activation in the olfactory system may also be a mechanism of degeneration that is common to other tauopathies where olfactory dysfunction occurs. Tau aggregates occur abundantly alongside alpha-synuclein pathology in the AON of cases with PD and Dementia with Lewy bodies [30]. Olfactory deficits are also an early and prevalent symptom in these diseases (reviewed by [36]). In other tauopathies such as PSP, corticobasal degeneration, ALS and FTD, olfactory symptoms are prevalent but mild, and tau pathology occurs throughout the brain yet there is very little or none in the olfactory bulb [36, 46]. Post-mortem studies have demonstrated that UPR activation occurs throughout the brain in these diseases including PSP, PD, ALS, and FTD variants with tau pathology [17, 22, 32, 43]. In our study, we noted that neurologically normal cases with Braak stage I-II show low levels of tau load and UPR activation in the olfactory regions, whereas those Braak stage 0 cases did not show any tau or UPR activation. Thus, UPR activation appears to occur in diseases with concomitant tau pathology and diseases where tau accumulation occurs in the olfactory bulb tend to show a greater severity of olfactory deficits. Therefore, it would be interesting to investigate whether UPR activation in the olfactory bulb is a common process in these diseases and if the amount of activation is relative to the AON tau burden in each disease.

In conclusion, we have demonstrated that UPR activation occurs in the olfactory system of AD cases with low-level tau pathology, indicative of the early disease stage. Within the olfactory bulb, UPR activation predominantly occurred within the anterior olfactory nucleus which is heavily affected by tau and beta-amyloid pathology from the earliest stages of AD. Together, these data indicate that UPR activation in the olfactory system may contribute to the earliest changes in AD.

## Supplementary information

**Additional file 1.** Correlation between percentage of p-PERK+ or p-eIF2α + neurons with age at death and post-mortem delay

**Additional file 2.** Distribution of p-PERK+ and p-eIF2α + neurons relative to beta-amyloid plaques in the AON. Immunohistochemistry for p-IRE1α and CK1δ in the AON and CA1 region.

**Additional file 3.** Summary of quantitative results and statistics

**Additional file 4.** Correlation between percentage of p-PERK+ and p-eIF2α + neurons and percentage of tau+ neurons in AD cases. Graph of p-PERK+ and p-eIF2α + cell density in regions of the human olfactory system in normal and AD cases.

## Data Availability

The data used for this study is available from the corresponding author on reasonable request.

## Abbreviations

AD: Alzheimer’s disease
ALS: Amyotrophic lateral sclerosis
AON: Anterior olfactory nucleus
ATF4: Activating transcription factor 4
ATF6: Activating transcription factor 6
BiP/GRP78: Binding immunoglobulin protein
CHOP: C/EBP-homologous protein
eIF2α: Eukaryotic Initiation Factor 2α
ER: Endoplasmic reticulum
FTD: Frontotemporal dementia
IRE1α: Inositol-requiring enzyme 1α
NFT: Neurofibrillary tangle
PD: Parkinson’s disease
PERK: Protein kinase R (PKR)-like endoplasmic reticulum kinase
PGP9.5: Protein Gene Product 9.5 (also known as ubiquitin C-terminal hydrolase 1. (UCHL-1))
PSP: Progressive supranuclear palsy
UPR: Unfolded protein response

## References

[1] Abisambra JF, Jinwal UK, Blair LJ, O’Leary JC, Li Q, Brady S, Wang L, Guidi CE, Zhang B, Nordhues BA, Cockman M, Suntharalingham A, Li P, Jin Y, Atkins CA, Dickey CA (2013). Tau accumulation activates the unfolded protein response by impairing endoplasmic reticulum-associated degradation. J Neurosci.

[2] Alcantarilla PF, Nuevo J, Bartoli A (2013). BMVC 2013 - Electron Proc Br Mach Vis Conf 2013.

[3] Allen Institute for Brain Science (2016). Allen human brain atlas.

[4] Attems J, Lintner F, Jellinger KA (2005). Olfactory involvement in aging and Alzheimer’s disease: an autopsy study. J Alzheimers Dis.

[5] Bhatnagar KP, Kennedy RC, Baron G, Greenberg RA (1987). Number of mitral cells and the bulb volume in the aging human olfactory bulb: a quantitative morphological study. Anat Rec.

[6] Braak H, Braak E (1995). Staging of Alzheimer’s disease-related neurofibrillary changes. Neurobiol Aging.

[7] Braak H, Del Tredici K, Bratzke H, Hamm-Clement J, Sandmann-Keil D, Rüb U (2002). Staging of the intracerebral inclusion body pathology associated with idiopathic Parkinson’s disease (preclinical and clinical stages). J Neurol.

[8] Braak H, Del Tredici K, Rüb U, de Vos RA, Jansen Steur EN, Braak E (2003). Staging of brain pathology related to sporadic Parkinson’s disease. Neurobiol Aging.

[9] Chafekar SM, Hoozemans JJM, Zwart R, Baas F, Scheper W (2007). Abeta 1-42 induces mild endoplasmic reticulum stress in an aggregation state-dependent manner. Antioxid Redox Signal.

[10] Cheng J, North BJ, Zhang T, Dai X, Tao K, Guo J, Wei W (2018). The emerging roles of protein homeostasis-governing pathways in Alzheimer’s disease. Aging Cell.

[11] Cornejo VH, Hetz C (2013). The unfolded protein response in Alzheimer’s disease. Semin Immunopathol.

[12] Devanand DP, Michaels-Marston KS, Liu X, Pelton GH, Padilla M, Marder K, Bell K, Stern Y, Mayeux R (2000). Olfactory deficits in patients with mild cognitive impairment predict Alzheimer’s disease at follow-up. Am J Psychiatry.

[13] Ding S-L, Royall JJ, Sunkin SM, Ng L, Facer BAC, Lesnar P, Guillozet-Bongaarts A, McMurray B, Szafer A, Dolbeare TA, Stevens A, Tirrell L, Benner T, Caldejon S, Dalley RA, Dee N, Lau C, Nyhus J, Reding M, Riley ZL, Sandman D, Shen E, van der Kouwe A, Varjabedian A, Wright M, Zöllei L, Dang C, Knowles JA, Koch C, Phillips JW, Sestan N, Wohnoutka P, Zielke HR, Hohmann JG, Jones AR, Bernard A, Hawrylycz MJ, Hof PR, Fischl B, Lein ES (2016). Comprehensive cellular-resolution atlas of the adult human brain. J Comp Neurol.

[14] Esiri MM, Wilcock GK (1984). The olfactory bulbs in Alzheimer’s disease. J Neurol Neurosurg Psychiatry.

[15] Guo T, Dakkak D, Rodriguez-Martin T, Noble W, Hanger DP (2019). A pathogenic tau fragment compromises microtubules, disrupts insulin signaling and induces the unfolded protein response. Acta Neuropathol Commun.

[16] Hetz C, Mollereau B (2014). Disturbance of endoplasmic reticulum proteostasis in neurodegenerative diseases. Nat Rev Neurosci.

[17] Hoozemans JJM, van Haastert ES, Eikelenboom P, de Vos RAI, Rozemuller JM, Scheper W (2007). Activation of the unfolded protein response in Parkinson’s disease. Biochem Biophys Res Commun.

[18] Hoozemans JJM, van Haastert ES, Nijholt DAT, Rozemuller AJM, Eikelenboom P, Scheper W (2009). The unfolded protein response is activated in pretangle neurons in Alzheimer’s disease hippocampus. Am J Pathol.

[19] Hoozemans JJM, van Haastert ES, Nijholt DAT, Rozemuller AJM, Scheper W (2012). Activation of the unfolded protein response is an early event in Alzheimer’s and Parkinson’s disease. Neurodegener Dis.

[20] Hoozemans JJM, Veerhuis R, Van Haastert ES, Rozemuller JM, Baas F, Eikelenboom P, Scheper W (2005). The unfolded protein response is activated in Alzheimer’s disease. Acta Neuropathol.

[21] Humphrey T, Crosby E (1938). The human olfactory bulb. Univ Hosp Bull.

[22] Ilieva EV, Ayala V, Jové M, Dalfó E, Cacabelos D, Povedano M, Bellmunt MJ, Ferrer I, Pamplona R, Portero-Otín M (2007). Oxidative and endoplasmic reticulum stress interplay in sporadic amyotrophic lateral sclerosis. Brain.

[23] Kovács T, Cairns NJ, Lantos PL (2001). Olfactory centres in Alzheimer’s disease: olfactory bulb is involved in early Braak’s stages. Neuroreport.

[24] ter Laak HJ, Renkawek K, van Workum FP (1994). The olfactory bulb in Alzheimer disease: a morphologic study of neuron loss, tangles, and senile plaques in relation to olfaction. Alzheimer Dis Assoc Disord.

[25] Lee DY, Lee K-S, Lee HJ, Kim DH, Noh YH, Yu K, Jung H-Y, Lee SH, Lee JY, Youn YC, Jeong Y, Kim DK, Lee WB, Kim SS (2010). Activation of PERK signaling attenuates Abeta-mediated ER stress. PLoS One.

[26] Lin L, Cao J, Yang S-S, Fu Z-Q, Zeng P, Chu J, Ning L-N, Zhang T, Shi Y, Tian Q, Zhou X-W, Wang J-Z (2018). Endoplasmic reticulum stress induces spatial memory deficits by activating GSK-3. J Cell Mol Med.

[27] Maresh A, Rodriguez Gil D, Whitman MC, Greer CA (2008) Principles of glomerular Organization in the Human Olfactory Bulb – implications for odor processing. PLoS One 3. 10.1371/journal.pone.0002640 10.1371/journal.pone.0002640 PMC2440537 18612420

[28] Meisami E, Mikhail L, Baim D, Bhatnagar KP (1998). Human olfactory bulb: aging of glomeruli and mitral cells and a search for the accessory olfactory bulb. Ann N Y Acad Sci.

[29] Moreno JA, Radford H, Peretti D, Steinert JR, Verity N, Martin MG, Halliday M, Morgan J, Dinsdale D, Ortori CA, Barrett DA, Tsaytler P, Bertolotti A, Willis AE, Bushell M, Mallucci GR (2012). Sustained translational repression by eIF2α-P mediates prion neurodegeneration. Nature.

[30] Mundiñano IC, Caballero MC, Ordóñez C, Hernandez M, DiCaudo C, Marcilla I, Erro ME, Tuñon MT, Luquin MR (2011). Increased dopaminergic cells and protein aggregates in the olfactory bulb of patients with neurodegenerative disorders. Acta Neuropathol.

[31] Murray HC, Swanson MEV, Dieriks BV, Turner C, Faull RLM, Curtis MA (2018) Neurochemical characterization of PSA-NCAM^+^cells in the human brain and phenotypic quantification in Alzheimer’s disease Entorhinal cortex. Neuroscience 372. 10.1016/j.neuroscience.2017.12.019 10.1016/j.neuroscience.2017.12.019 29429526

[32] Nijholt DA, van Haastert ES, AJM R, Scheper W, JJM H (2012). The unfolded protein response is associated with early tau pathology in the hippocampus of tauopathies. J Pathol.

[33] O’Connor T, Sadleir KR, Maus E, Velliquette RA, Zhao J, Cole SL, Eimer WA, Hitt B, Bembinster LA, Lammich S, Lichtenthaler SF, Hébert SS, De Strooper B, Haass C, Bennett DA, Vassar R (2008). Phosphorylation of the translation initiation factor eIF2α increases BACE1 levels and promotes Amyloidogenesis. Neuron.

[34] Pearce RKB, Hawkes CH, Daniel SE (1995). The anterior olfactory nucleus in Parkinson’s disease. Mov Disord.

[35] Resende R, Ferreiro E, Pereira C, Oliveira CR (2008). ER stress is involved in Abeta-induced GSK-3beta activation and tau phosphorylation. J Neurosci Res.

[36] Rey NL, Wesson DW, Brundin P (2016). The olfactory bulb as the entry site for prion-like propagation in neurodegenerative diseases. Neurobiol Dis.

[37] Sánchez DS, Bañón IU, de la Rosa PC, Palacios L, Muñozguren SG, Insausti R, Marcos AM (2010). The human olfactory system: an anatomical and cytoarchitectonic study of the anterior olfactory nucleus.

[38] Saxena S, Caroni P (2011). Selective neuronal vulnerability in neurodegenerative diseases: from stressor thresholds to degeneration. Neuron.

[39] Scheper W, Hoozemans JJM (2013). A new PERKspective on neurodegeneration. Sci Transl med.

[40] Scheper W, Hoozemans JJM (2015). The unfolded protein response in neurodegenerative diseases: a neuropathological perspective. Acta Neuropathol.

[41] Sengoku R, Saito Y, Ikemura M, Hatsuta H (2008). Incidence and Extent of Lewy Body-Related a-Synucleinopathy in Aging Human Olfactory Bulb. J Neuropathol Exp Neurol.

[42] Stevenson TJ, Murray HC, Turner C, Faull RLM, Dieriks BV, Curtis MA (2020) α -synuclein inclusions are abundant in non-neuronal cells in the anterior olfactory nucleus of the Parkinson’s disease olfactory bulb. Sci Rep:1–10. 10.1038/s41598-020-63412-x 10.1038/s41598-020-63412-x PMC7174302 32317654

[43] Stutzbach LD, Xie SX, Naj AC, Albin R, Gilman S, VMY L, Trojanowski JQ, Devlin B, Schellenberg GD, PSP Genetics Study Group (2013). The unfolded protein response is activated in disease-affected brain regions in progressive supranuclear palsy and Alzheimer’s disease. Acta Neuropathol Commun.

[44] Tabas I, Ron D (2011). Integrating the mechanisms of apoptosis induced by endoplasmic reticulum stress. Nat Cell Biol.

[45] Takeda T, Iijima M, Uchihara T, Ohashi T, Seilhean D, Duyckaerts C, Uchiyama S (2015). TDP-43 pathology progression along the olfactory pathway as a possible substrate for olfactory impairment in amyotrophic lateral sclerosis. J Neuropathol Exp Neurol.

[46] Tsuboi Y, Wszolek ZK, Graff-Radford NR, Cookson N, Dickson DW (2003). Tau pathology in the olfactory bulb correlates with Braak stage, Lewy body pathology and apolipoprotein epsilon4. Neuropathol Appl Neurobiol.

[47] Unterberger U, Höftberger R, Gelpi E, Flicker H, Budka H, Voigtländer T (2006). Endoplasmic reticulum stress features are prominent in Alzheimer disease but not in prion diseases in vivo. J Neuropathol Exp Neurol.

[48] Waldvogel HJ, Curtis MA, Baer K, Rees MI, RLM F (2007). Immunohistochemical staining of post-mortem adult human brain sections. Nat Protoc.

[49] Wiersma VI, van Ziel AM, Vazquez-Sanchez S, Nölle A, Berenjeno-Correa E, Bonaterra-Pastra A, Clavaguera F, Tolnay M, Musters RJP, van Weering JRT, Verhage M, Hoozemans JJM, Scheper W (2019). Granulovacuolar degeneration bodies are neuron-selective lysosomal structures induced by intracellular tau pathology. Acta Neuropathol.

[50] Zapiec B, Dieriks BV, Tan S, Faull RLM, Mombaerts P, Curtis MA (2017). A ventral glomerular deficit in Parkinson’s disease revealed by whole olfactory bulb reconstruction. Brain.

