# Supplementary material for: The unfolded protein response is activated in the olfactory system in Alzheimer’s disease
Source: Acta Neuropathol Commun. 2020 Jul 14;8:109. doi: 10.1186/s40478-020-00986-7 (PMC7362534; doi:10.1186/s40478-020-00986-7)
Supplement: Supplementary file 1 — Additional file 1. Correlation between percentage of p-PERK+ or p-eIF2α + neurons with age at death and post-mortem delay [file 40478_2020_986_MOESM1_ESM.docx]

**Additional File 1.**

Supplementary Figure 1. Correlation between percentage of p-PERK+ or p-eIF2α+ neurons with age at death and post-mortem delay.

**
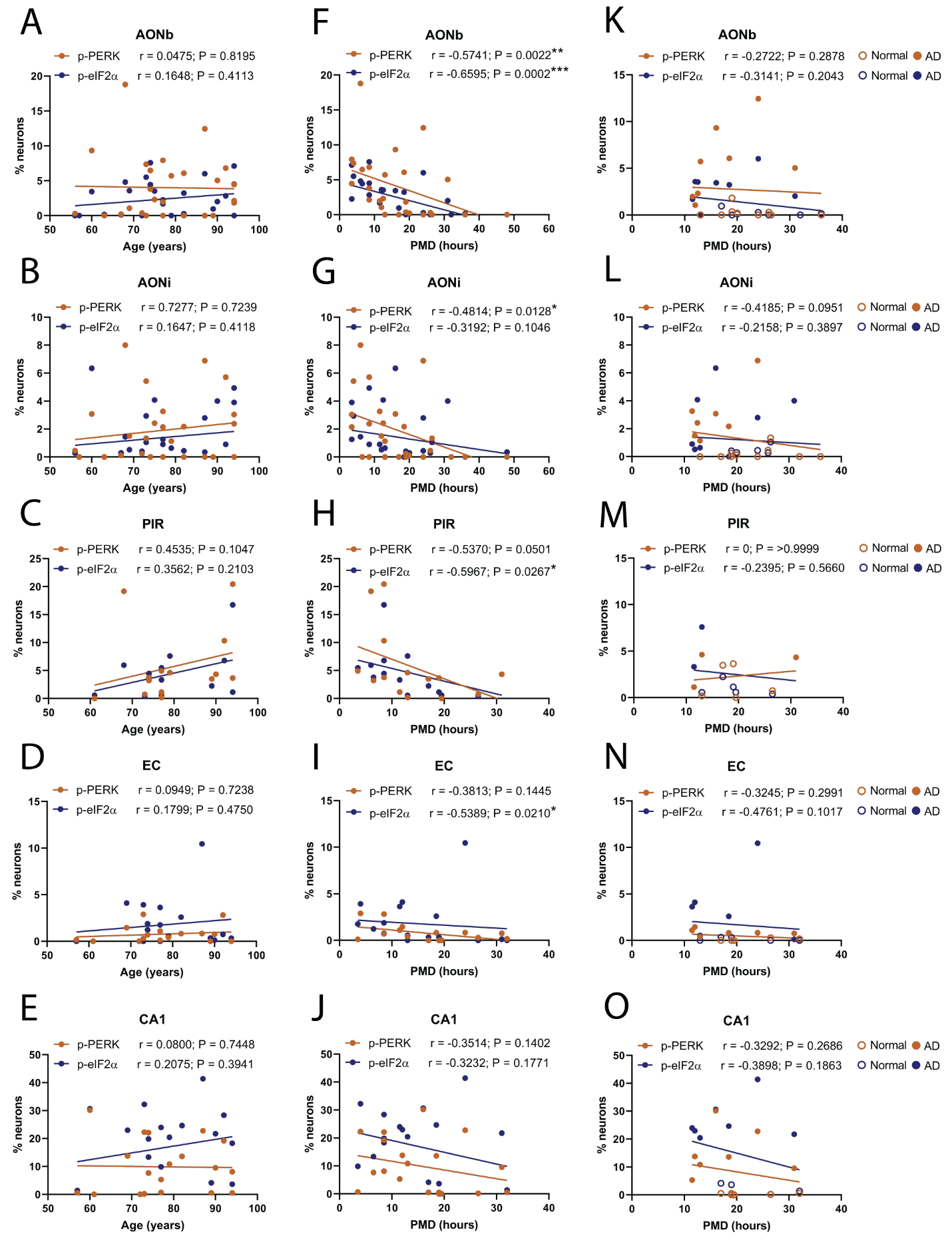
**

**Supplementary Figure 1. Correlation between percentage of** **p-PERK+ or p-eIF2α+ neurons with age at death and post-mortem delay.** For each region, the percentage of p-PERK+ or p-eIF2α+ neurons was plotted against age at death (A-E) and post-mortem delay (F-J) for each case. A non-parametric Spearman’s correlation test determined that there was no significant correlation between the percentage of p-PERK+ or p-eIF2α+ neurons and age at death in any of the regions assessed. There was also no significant correlation between the percentage of p-PERK+ or p-eIF2α+ neurons and post-mortem delay in the CA1 region. However, there were moderate inverse correlations between the percentage of p-PERK+ neurons and post-mortem delay in the AONb and AONi and between p-eIF2α+ neurons and post-mortem delay in the AONb, PIR and EC regions. As this is likely due to the difference in post-mortem delay between normal and AD cases, we investigated a partial correlation by plotting cases with a post-mortem delay between 11.5 and 36 hours (K-O). This range in post-mortem delay overlapped between the normal and AD groups and no significant correlation was observed for either p-PERK or p-eIF2α in any region. Abbreviations: PMD, post-mortem delay.
