# Supplementary material for: The unfolded protein response is activated in the olfactory system in Alzheimer’s disease
Source: Acta Neuropathol Commun. 2020 Jul 14;8:109. doi: 10.1186/s40478-020-00986-7 (PMC7362534; doi:10.1186/s40478-020-00986-7)
Supplement: Supplementary file 2 — Additional file 2. Distribution of p-PERK+ and p-eIF2α + neurons relative to beta-amyloid plaques in the AON. Immunohistochemistry for p-IRE1α and CK1δ in the AON and CA1 region. [file 40478_2020_986_MOESM2_ESM.docx]

**Additional File 2.**

Supplementary Figure 2. Distribution of p-PERK+ and p-eIF2α+ cells and beta-amyloid plaques in the AON.

Supplementary Figure 3. Fluorescent immunohistochemistry for p-IRE1α in the AON and CA1 region.


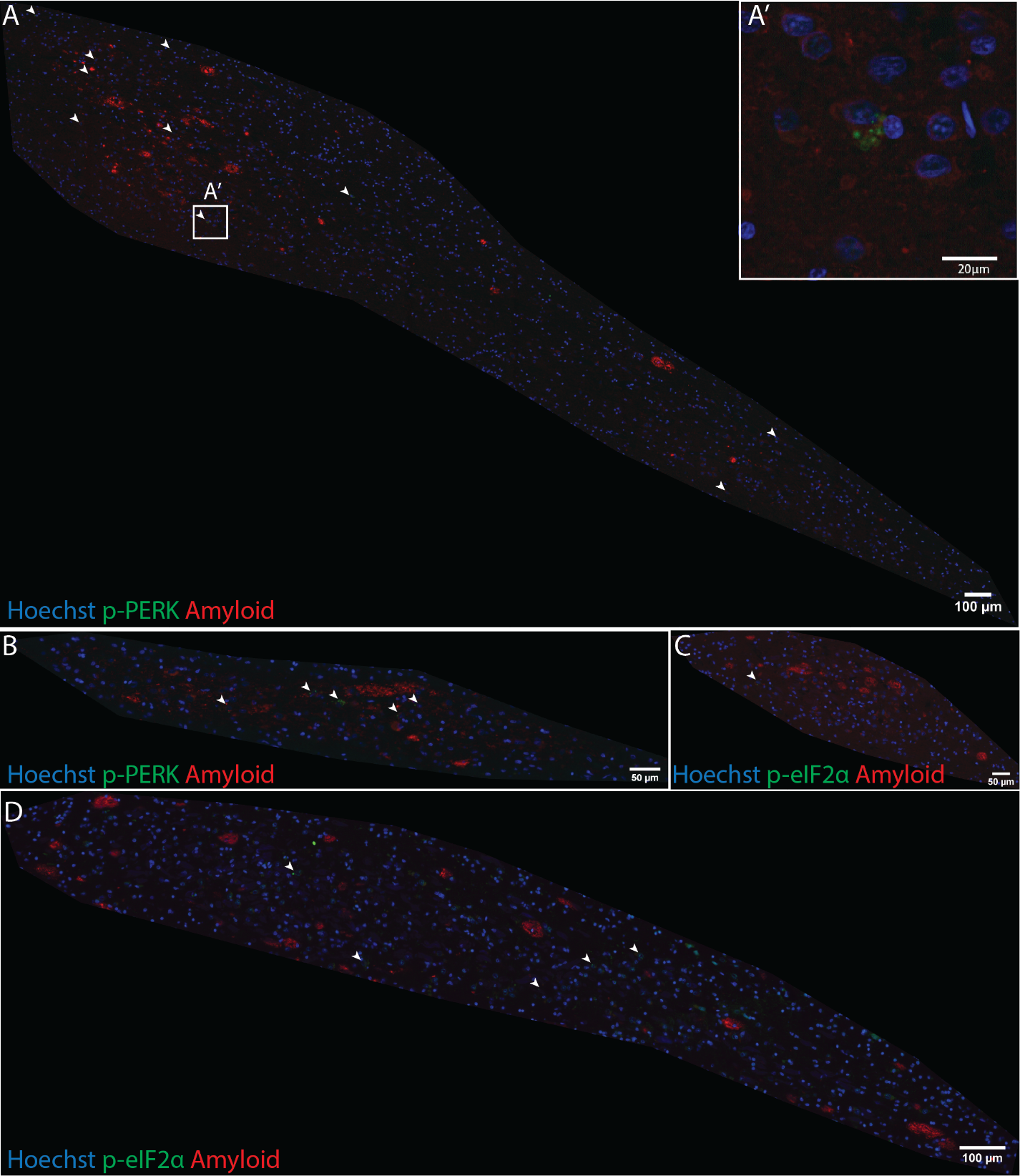


**Supplementary Figure 2. Distribution of p-PERK+ or p-eIF2+ cells and beta-amyloid plaques in the AON.** AON region of interest images from representative AD cases indicating the location of p-PERK+ (A-B) and p-eIF2α+ (C–D) cells (white arrows) relative to beta-amyloid plaques (red). No spatial relationship was qualitatively observed within the AON.

**
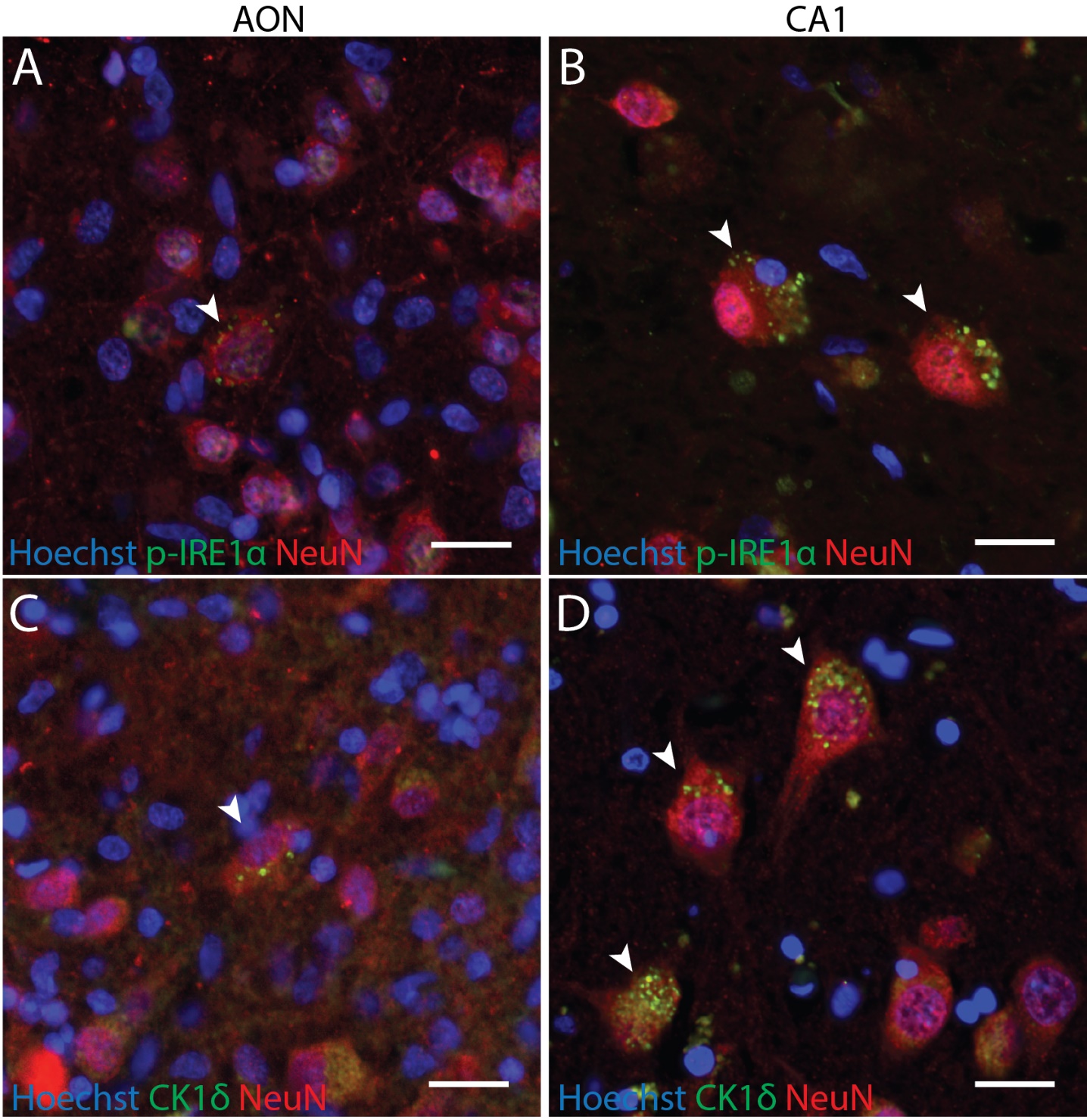
**

**Supplementary Figure 3. Fluorescent immunohistochemistry for p-IRE1α and CK1δ in the AON and CA1 region.** Some p-IRE1α+ neurons were detected in the AON (A), but p-IRE1α+ neurons were far more abundant in the CA1 region (B). CK1δ immunoreactivity was observed within AON (C) and CA1 (D) neurons. Arrows indicate p-IRE1α+ neurons. Scale bars = 20 μm.
