# Supplementary material for: The unfolded protein response is activated in the olfactory system in Alzheimer’s disease
Source: Acta Neuropathol Commun. 2020 Jul 14;8:109. doi: 10.1186/s40478-020-00986-7 (PMC7362534; doi:10.1186/s40478-020-00986-7)
Supplement: Supplementary file 3 — Additional file 3. Summary of quantitative results and statistics [file 40478_2020_986_MOESM3_ESM.docx]

## Additional File 3

Supplementary Table 1. Summary of p-PERK and p-eIF2α quantitative results (mean ± SD)

Supplementary Table 2. Summary of p-values for between regions analysis of p-PERK and p-eIF2α (AD cases only)

Supplementary Table 3: Summary of tau and amyloid load quantitative results (mean ± SD)

Supplementary Table 4: Summary of p-values for the comparison of tau load and amyloid load between regions. (AD cases only)

Supplementary Table 5: Summary of p-values for Braak stage analysis of % p-PERK+, % p-eIF2α+ and % tau+ neurons

Supplementary Table 6. Summary of p-PERK, p-eIF2α and tau co-labeling results (mean ± SD)

Supplementary Table 7. Summary of p-values for p-PERK, p-eIF2α and tau co-labelling results

Supplementary Table 1. Summary of p-PERK and p-eIF2α quantitative results (mean ± SD)

| p-PERK (% p-PERK+ neurons) | | | |
| --- | --- | --- | --- |
| Region | Mean of Normal Cases | Mean of AD Cases | P-value |
| AONb | 0.2665 ± 0.5624 | 6.352 ± 4.459 | <0.0001**** |
| AONi | 0.1958 ± 0.4062 | 3.145 ± 2.375 | 0.0002*** |
| PIR | 1.611 ± 1.807 | 7.991 ± 7.139 | 0.0120* |
| EC | 0.0072 ± 0.1257 | 1.189 ± 0.9499 | 0.001*** |
| CA1 | 0.2914 ± 0.2709 | 14.30 ± 8.446 | 0.0009*** |
| p-eIF2α (% p-eIF2α+ neurons) | | | |
| Region | Mean of Normal Cases | Mean of AD Cases | P-value |
| AONb | 0.1378 ± 0.2873 | 3.908 ± 2.012 | <0.0001**** |
| AONi | 0.2581 ± 0.3203 | 2.169 ± 1.998 | 0.0025** |
| PIR | 0.9845 ± 0.7600 | 6.488 ± 4.091 | 0.0010*** |
| EC | 0.0966 ± 0.1650 | 2.816 ± 2.886 | 0.0003*** |
| CA1 | 1.559 ± 1.882 | 23.67 ± 8.244 | <0.0001**** |

Supplementary Table 2. Summary of p-values for between regions analysis of p-PERK and p-eIF2α (AD cases only)

| % p-PERK+ neurons | | | | | | |  |  |
| --- | --- | --- | --- | --- | --- | --- | --- | --- |
|  | AONb | | AONi | | PIR | | EC | CA1 |
| AONb |  | | 0.6944 | | >0.9999 | | 0.0083** | 0.3629 |
| AONi |  | |  | | 0.8118 | | 0.8883 | 0.0015** |
| PIR |  | |  | |  | | 0.0186* | >0.9999 |
| EC |  | |  | |  | |  | <0.0001**** |
| CA1 |  | |  | |  | |  |  |
| % p-eIF2α+ neurons | | | | | |  |  |  |
|  | | AONb | | AONi | | PIR | EC | CA1 |
| AONb | |  | | 0.8534 | | >0.9999 | >0.9999 | 0.0005*** |
| AONi | |  | |  | | 0.052 | >0.9999 | <0.0001**** |
| PIR | |  | |  | |  | 0.2247 | 0.2723 |
| EC | |  | |  | |  |  | <0.0001**** |
| CA1 | |  | |  | |  |  |  |

Supplementary Table 3: Summary of tau and amyloid load quantitative results (mean ± SD)

| Tau Load (% area of image stained) | | | |
| --- | --- | --- | --- |
| Region | Mean of Normal Cases | Mean of AD Cases | P-value |
| GL | 0.0146 ± 0.0217 | 0.1264 ± 0.1261 | 0.0002*** |
| ML | 0.0227 ± 0.0251 | 0.3839 ± 0.2765 | <0.0001**** |
| AONb | 0.1417 ± 0.3167 | 3.819 ± 2.701 | <0.0001**** |
| AONi | 0.0455 ± 0.0553 | 2.821 ± 2.397 | <0.0001**** |
| PIR | 0.1621 ± 0.1705 | 1.447 ± 1.160 | 0.005*** |
| EC | 0.0232 ± 0.0370 | 0.6943 ± 0.7162 | 0.0003*** |
| CA1 | 0.0954 ± 0.1333 | 1.833 ± 1.862 | 0.009*** |
| Beta-Amyloid Load (% area of image stained) | | | |
| Region | Mean of Normal Cases | Mean of AD Cases | P-value |
| GL | 0.0112 ± 0.0178 | 0.0703 ± 0.0767 | 0.0159* |
| ML | 0.0101 ± 0.0093 | 0.0703 ± 0.0555 | 0.0015** |
| AONb | 0.0365 ± 0.0751 | 1.209 ± 0.9320 | <0.0001**** |
| AONi | 0.03581 ± 0.0406 | 0.0852 ± 0.0870 | 0.2494 |
| PIR | 0.0722 ± 0.0545 | 0.6274 ± 0.3940 | 0.0082** |
| EC | 0.0918 ± 0.1195 | 1.175 ± 1.000 | 0.0018** |
| CA1 | 0.1851 ± 0.3025 | 0.6015 ± 0.5262 | 0.0285* |

Supplementary Table 4: Summary of p-values for the comparison of tau load and amyloid load between regions. (AD cases only)

| Tau Load (% area of image stained) | | | | |  |  |  |  |
| --- | --- | --- | --- | --- | --- | --- | --- | --- |
|  | GL | ML | | AONb | AONi | PIR | EC | CA1 |
| GL |  | >0.9999 | | <0.0001**** | <0.0001**** | 0.0028** | 0.7791 | 0.0007*** |
| ML |  |  | | <0.0001**** | 0.0014** | 0.3141 | >0.9999 | 0.1535 |
| AONb |  |  | |  | >0.9999 | >0.9999 | 0.0034 | >0.9999 |
| AONi |  |  | |  |  | >0.9999 | 0.0349* | >0.9999 |
| PIR |  |  | |  |  |  | >0.9999 | >0.9999 |
| EC |  |  | |  |  |  |  | >0.9999 |
| CA1 |  |  | |  |  |  |  |  |
| Amyloid Load (% area of image stained) | | | | |  |  |  |  |
|  | GL | | ML | AONb | AONi | PIR | EC | CA1 |
| GL |  | | >0.9999 | <0.0001**** | >0.9999 | 0.0062** | 0.0004*** | 0.0346* |
| ML |  | |  | 0.0002*** | >0.9999 | 0.0108* | 0.0007*** | 0.0578 |
| AONb |  | |  |  | <0.0001 | >0.9999 | >0.9999 | >0.9999 |
| AONi |  | |  |  |  | 0.0073** | 0.0004*** | 0.042* |
| PIR |  | |  |  |  |  | >0.9999 | >0.9999 |
| EC |  | |  |  |  |  |  | >0.9999 |
| CA1 |  | |  |  |  |  |  |  |

Supplementary Table 5: Summary of p-values for Braak stage analysis of % p-PERK+, % p-eIF2α+ and % tau+ neurons

| % p-PERK+ neurons | | | | |  | % p-eIF2α+ neurons | | | | |  | % tau+ neurons | | | | |
| --- | --- | --- | --- | --- | --- | --- | --- | --- | --- | --- | --- | --- | --- | --- | --- | --- |
| AONb | 0 | I-II | III-IV | V-VI |  | AONb | 0 | I-II | III-IV | V-VI |  | AONb | 0 | I-II | III-IV | V-VI |
| 0 |  | >0.9999 | 0.1925 | 0.2052 |  | 0 |  | >0.9999 | 0.4593 | 0.0139* |  | 0 |  | >0.9999 | 0.0532 | 0.0240* |
| I-II |  |  | 0.0923 | 0.1001 |  | I-II |  |  | 0.7892 | 0.0332* |  | I-II |  |  | 0.8016 | 0.5109 |
| III-IV |  |  |  | >0.9999 |  | III-IV |  |  |  | 0.6517 |  | III-IV |  |  |  | >0.9999 |
| V-VI |  |  |  |  |  | V-VI |  |  |  |  |  | V-VI |  |  |  |  |
| AONi | 0 | I-II | III-IV | V-VI |  | AONi | 0 | I-II | III-IV | V-VI |  | AONi | 0 | I-II | III-IV | V-VI |
| 0 |  | >0.9999 | 0.2869 | 0.1846 |  | 0 |  | >0.9999 | >0.9999 | 0.0985 |  | 0 |  | >0.9999 | 0.0253* | 0.0500* |
| I-II |  |  | 0.4078 | 0.2749 |  | I-II |  |  | >0.9999 | 0.2677 |  | I-II |  |  | 0.0947 | 0.1778 |
| III-IV |  |  |  | >0.9999 |  | III-IV |  |  |  | 0.8674 |  | III-IV |  |  |  | >0.9999 |
| V-VI |  |  |  |  |  | V-VI |  |  |  |  |  | V-VI |  |  |  |  |
| PIR | 0 | I-II | III-IV | V-VI |  | PIR | 0 | I-II | III-IV | V-VI |  | PIR | 0 | I-II | III-IV | V-VI |
| 0 |  | >0.9999 | 0.3237 | 0.0457* |  | 0 |  | >0.9999 | 0.3335 | 0.1138 |  | 0 |  | >0.9999 | 0.1923 | 0.0373* |
| I-II |  |  | >0.9999 | 0.2992 |  | I-II |  |  | 0.3289 | 0.0985 |  | I-II |  |  | 0.4988 | 0.0942 |
| III-IV |  |  |  | >0.9999 |  | III-IV |  |  |  | >0.9999 |  | III-IV |  |  |  | >0.9999 |
| V-VI |  |  |  |  |  | V-VI |  |  |  |  |  | V-VI |  |  |  |  |
| EC | 0 | I-II | III-IV | V-VI |  | EC | 0 | I-II | III-IV | V-VI |  | EC | 0 | I-II | III-IV | V-VI |
| 0 |  | >0.9999 | 0.1144 | 0.2531 |  | 0 |  | >0.9999 | 0.0449* | 0.0125* |  | 0 |  | >0.9999 | 0.0777 | 0.0174* |
| I-II |  |  | 0.1488 | 0.3147 |  | I-II |  |  | 0.6489 | 0.2110 |  | I-II |  |  | >0.9999 | 0.5276 |
| III-IV |  |  |  | >0.9999 |  | III-IV |  |  |  | >0.9999 |  | III-IV |  |  |  | >0.9999 |
| V-VI |  |  |  |  |  | V-VI |  |  |  |  |  | V-VI |  |  |  |  |
| CA1 | 0 | I-II | III-IV | V-VI |  | CA1 | 0 | I-II | III-IV | V-VI |  | CA1 | 0 | I-II | III-IV | V-VI |
| 0 |  | >0.9999 | 0.0237* | 0.0009*** |  | 0 |  | >0.9999 | <0.0001**** | <0.0001**** | | 0 |  | >0.9999 | 0.0538 | 0.0153** |
| I-II |  |  | 0.0286* | 0.0011** |  | I-II |  |  | <0.0001**** | <0.0001**** | | I-II |  |  | 0.3944 | 0.1419 |
| III-IV |  |  |  | 0.9978 |  | III-IV |  |  |  | 0.9997 |  | III-IV |  |  |  | >0.9999 |
| V-VI |  |  |  |  |  | V-VI |  |  |  |  |  | V-VI |  |  |  |  |

Supplementary Table 6. Summary of p-PERK, p-eIF2α and tau co-labeling results (mean ± SD)

| Region | % p-PERK+ neurons with tau | % p-eIF2α+ neurons with tau | % tau+ neurons with p-PERK | % tau+ neurons with p-eIF2α |
| --- | --- | --- | --- | --- |
| AONb | 15.2 ± 14.0% | 12.5 ± 13.6% | 4.0 ± 4.2% | 2.0 ± 1.9% |
| AONi | 15.6 ± 27.0% | 5.7 ± 12.2% | 1.4 ± 2.1% | 0.60 ± 1.4% |
| EC | 8.6 ± 18.1% | 9.4 ± 17.5% | 0.62 ± 1.9% | 3.4 ± 5.6% |
| CA1 | 12.4 ± 11.1% | 7.0 ± 9.2% | 13.5 ± 13.0% | 7.9 ± 7.6% |

Supplementary Table 7. Summary of p-values for p-PERK, p-eIF2α and tau co-labelling results

| % p-PERK+ neurons with tau | | | | |
| --- | --- | --- | --- | --- |
|  | AONb | AONi | EC | CA1 |
| AONb |  | >0.9999 | 0.4902 | >0.9999 |
| AONi |  |  | >0.9999 | >0.9999 |
| EC |  |  |  | >0.9999 |
| CA1 |  |  |  |  |
| % p-eIF2α+ neurons with tau | | | |  |
|  | AONb | AONi | EC | CA1 |
| AONb |  | 0.0625 | 0.8358 | >0.9999 |
| AONi |  |  | >0.9999 | >0.9999 |
| EC |  |  |  | >0.9999 |
| CA1 |  |  |  |  |
| % tau+ neurons with p-PERK | | | |  |
|  | AONb | AONi | EC | CA1 |
| AONb |  | 0.8006 | 0.0706 | 0.982 |
| AONi |  |  | >0.9999 | 0.0341* |
| EC |  |  |  | 0.002** |
| CA1 |  |  |  |  |
| % tau+ neurons with p-eIF2α | | | |  |
|  | AONb | AONi | EC | CA1 |
| AONb |  | 0.1389 | >0.9999 | >0.9999 |
| AONi |  |  | >0.9999 | 0.0088** |
| EC |  |  |  | 0.5113 |
| CA1 |  |  |  |  |
