# Supplementary material for: The unfolded protein response is activated in the olfactory system in Alzheimer’s disease
Source: Acta Neuropathol Commun. 2020 Jul 14;8:109. doi: 10.1186/s40478-020-00986-7 (PMC7362534; doi:10.1186/s40478-020-00986-7)
Supplement: Supplementary file 4 — Additional file 4. Correlation between percentage of p-PERK+ and p-eIF2α + neurons and percentage of tau+ neurons in AD cases. Graph of p-PERK+ and p-eIF2α + cell density in regions of the human olfactory system in normal and AD cases. [file 40478_2020_986_MOESM4_ESM.docx]

## Additional File 4

Supplementary Figure 4. Correlation between percentage of p-PERK+ and p-eIF2α+ neurons and percentage of tau+ neurons in AD cases.

Supplementary Figure 5. Quantification of p-PERK+ and p-eIF2α+ cell density in regions of the human olfactory system in normal and AD cases.

**
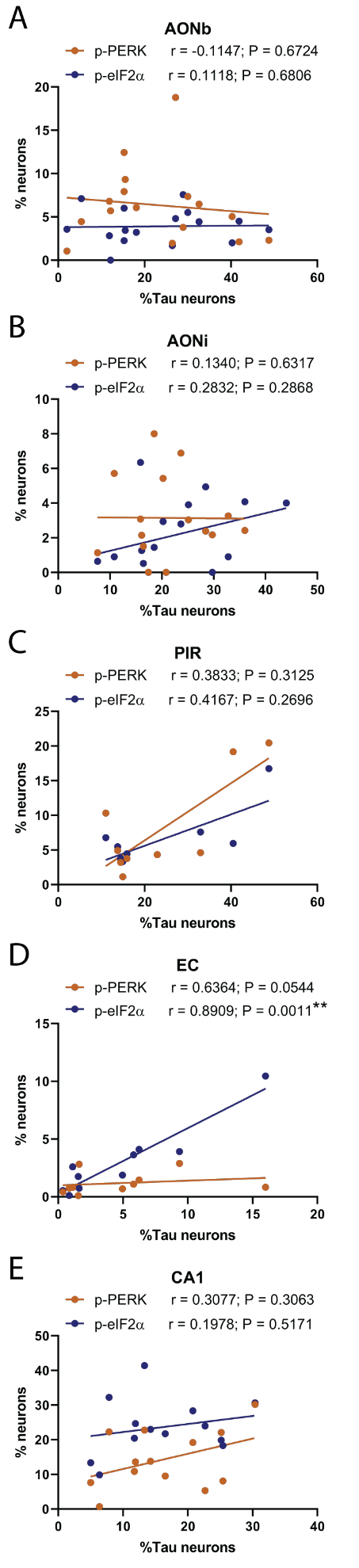
Supplementary Figure 4. Correlation between percentage of p-PERK+ and p-eIF2α+ neurons and percentage of tau+ neurons in AD cases.** For each region the percentage of p-PERK+ or p-eIF2α+ neurons were plotted against the percentage of tau+ neurons for each case. A non-parametric Spearman’s correlation test determined that there were no significant correlations in the AONb, AONi, piriform cortex or CA1 region, however there was a significant positive correlation between the percentage of p-eIF2α+ neurons and tau+ neurons in the entorhinal cortex.


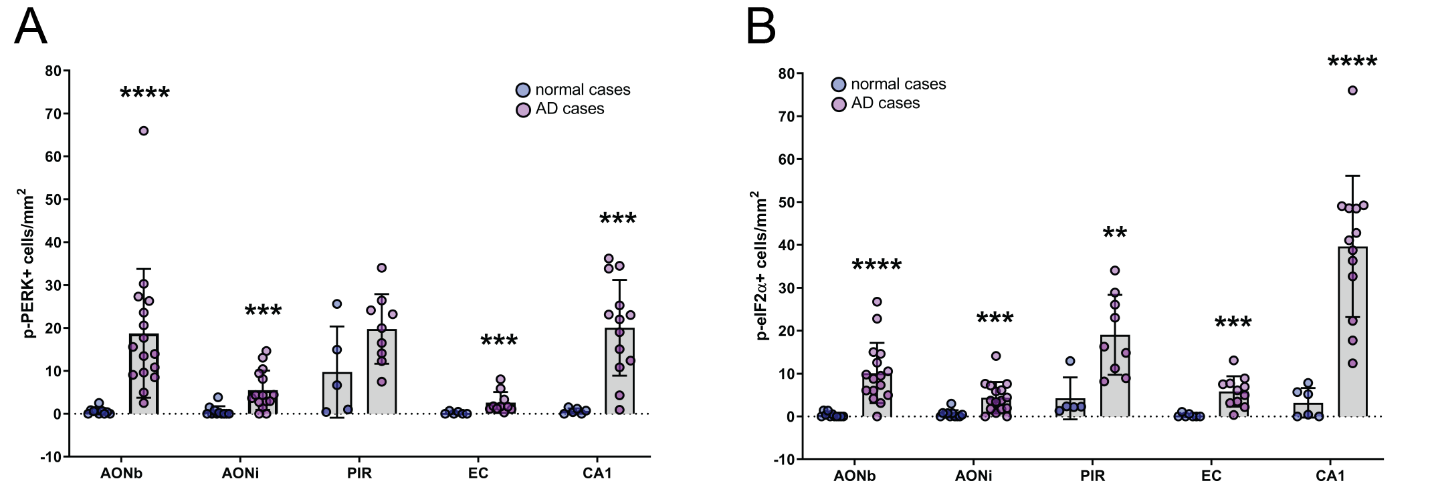


**Supplementary Figure 5.** **Quantification of p-PERK+ and p-eIF2α+ cell density in regions of the human olfactory system in normal and AD cases.** The density of total p-PERK+ and p-eIF2α+ cells in each region was measured as p-PERK+ (A) and p-eIF2α+ (B) cells per mm^2^. The density of p-PERK+ and p-eIF2α+ cells was significantly increased in AD cases compared to normals for all regions assessed, except for p-PERK in the piriform cortex. **P ≤ 0.01, ***P ≤ 0.001, ****P ≤ 0.0001 compared to normals in the same region.
